# How People with Parkinson's Disease and Health Care Professionals Wish to Partner in Care Using eHealth: Co-Design Study

**DOI:** 10.2196/19195

**Published:** 2020-09-21

**Authors:** Carolina Wannheden, Åsa Revenäs

**Affiliations:** 1 Medical Management Centre Department of Learning, Informatics, Management and Ethics (LIME) Karolinska Institutet Stockholm Sweden; 2 Centre for Clinical Research County of Västmanland Uppsala University Västerås Sweden; 3 School of Health, Care and Social Welfare Division of Physiotherapy Mälardalen University Västerås Sweden

**Keywords:** chronic care, Parkinson's disease, co-creation, co-design, participatory design, eHealth, mHealth, clinical decision support

## Abstract

**Background:**

Worldwide, the number of people with Parkinson’s disease (PD) is predicted to double between the years 2005 and 2030. Chronic care management requires active collaboration and knowledge exchange between patients and health care professionals (HCPs) for best possible health outcomes, which we describe as co-care. eHealth services have the potential to support the realization of co-care between people with PD (PwP) and HCPs.

**Objective:**

This study aimed to explore how co-care could be operationalized in PD care, supported by eHealth. More specifically, this study explores PwP's and HCPs' expectations and desired eHealth functionalities to achieve co-care.

**Methods:**

Principles of participatory design were used to enable the identification of co-care needs and design ideas, in a series of 4 half-day co-design workshops. The sample included 7 (4 women) PwP and 9 (4 women) HCPs, including 4 neurologists, 3 nurses, and 2 physiotherapists. The co-design process resulted in a functional prototype that was evaluated by the co-design participants in the last workshop. Data were collected through note cards produced by the participants during the first 3 workshops and focus group discussions during the 3rd and 4th workshops. The data were analyzed using qualitative thematic analysis. After the workshop series, the prototype was demonstrated at a Mini Fair for ongoing PD research and evaluated using a self-developed questionnaire with 37 respondents: 31 PwP (14 women) and 6 informal caregivers (3 women). Descriptive statistics are reported.

**Results:**

The qualitative analysis of data resulted in 2 main themes. The first theme, core eHealth functionalities and their expected values, describes 6 desired eHealth functionalities for supporting PD co-care between PwP and HCPs: (1) self-tracking, (2) previsit forms, (3) graphical visualization, (4) clinical decision support, (5) self-care recommendations, and (6) asynchronous communication. The second theme, individual and organizational constraints, describes constraints that need to be addressed to succeed with an eHealth service for co-care. Individual constraints include eHealth literacy and acceptance; organizational constraints include teamwork and administrative workload. The majority of the questionnaire respondents (31/37, 84%) perceived that they would benefit from an eHealth service similar to the demonstrated prototype. All prototype functionalities were rated as very important or important by the majority of respondents (ranging from 86% to 97% per functionality).

**Conclusions:**

This study adds to our knowledge on how PD co-care could be operationalized. Co-care implies a shift from episodic routine-driven care to more flexible care management that is driven by the mutual needs of patients and HCPs and supported by active information exchange between them, as well as automated information processing to generate patient-specific advice. More research is needed to further explore the concept of co-care in chronic care management and what it means for self-care and health care.

**International Registered Report Identifier (IRRID):**

RR2-10.2196/11278

## Introduction

Chronic conditions affect more than 80% of people aged over 65 years in the European Union and represent a major challenge for health and social care systems [1]. Parkinson's disease (PD) is the second most common neurodegenerative disorder following Alzheimer’s disease. It causes motor and nonmotor symptoms and results in significant burden for individual patients and their families, as well as health care and society [2]. Worldwide, the number of people with PD (PwP) is predicted to double between the years 2005 and 2030 [3]. Given this predicted increase and the limited availability of health care resources, self-management in everyday life is crucial for PwP as well as for people with other chronic conditions.

### Co-Care

Chronic disease management requires a different practice of health care compared to the management of acute conditions [4]. This practice emphasizes both patients’ and health care professionals’ (HCPs’) knowledge and active engagement for best possible health outcomes [5,6]. The term co-care, as defined by von Thiele Schwarz [7], emphasizes the use of appropriate tools, such as health information technologies, to enable the creation, shaping, sharing, and application of knowledge between different actors who are involved in an individual’s care.

### eHealth and e-Patients

eHealth refers to “health services and information delivered or enhanced through the internet and related technologies” [8]. The internet is an important resource for individuals with chronic conditions to acquire disease-specific knowledge [9] and also among PwP [10]. In 2004, Ferguson and Frydman [11] described patients and informal caregivers who sought online health guidance, for example through health communities, as the first generation of e-patients. Fifteen years later, the second generation of e-patients was described as patients who engage actively in their self-care and health care by producing and sharing their own health data as well as contributing to digital health innovations [12], which indicates a transition towards co-care.

### eHealth in Parkinson's Disease Care

There is considerable evidence indicating that eHealth can be effective or at least promising in somatic care [13]. Identified values include real-time monitoring, better tailored personalized services, and patient empowerment [14]. A great number of PD applications are available to support individuals with diagnosis-specific information, assessments, and treatment [15]. In particular, mobile health technologies can support remote monitoring of PD motor symptoms by use of wireless motor sensors [16-18]. Such technologies have been widely recognized as promising [19,20], but the clinical utility of the self-tracked data and their value to improve health care requires further research [17,21,22].

It has been suggested that eHealth tools that support direct patient-provider communication may be more effective at improving patient self-management and self-efficacy [23]. PD technologies still have a tendency to prioritize the physician’s perspective, while the needs of PwP and informal caregivers may not be fully supported [24]. To the best of our knowledge, how to enable PwP and HCPs to partner effectively in co-care, supported by eHealth, has not been described.

### Aim

The aim of this study was to explore how co-care could be operationalized in PD care, supported by eHealth. More specifically, this study explores PwP’s and HCPs’ expectations and desired eHealth functionalities to achieve co-care.

## Methods

### Study Design and Participants

Principles of participatory design were used to enable the identification of PwP’s and HCPs’ views and expectations on co-care [25]. Participatory design shares similarities with action research and offers a method for combining health service and technology development in close collaboration with the intended users of the future service [25].

#### Co-Design Workshops

**Figure 1:**
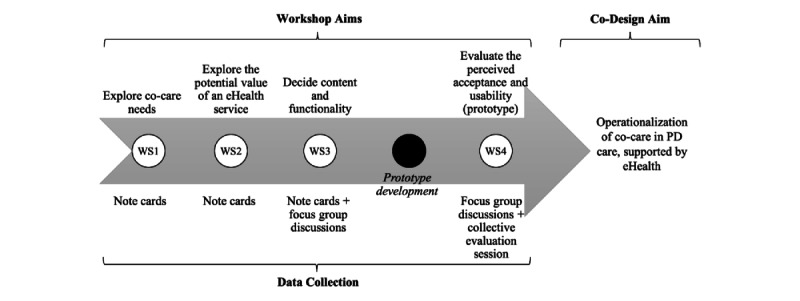
Overview of the co-design workshops performed to explore how people with Parkinson's disease (PD) and health care professionals would like to partner in chronic care management, with particular emphasis on how eHealth could support them. WS: workshop.

The collaborative work was performed in a series of 4 half-day co-design workshops during May and June of 2016 [26,27]. Participants in the co-design workshops included 7 PwP (4 women) and 9 HCPs (4 women). Among the HCPs, 3 were registered nurses, 4 were neurologists, and 2 were registered physiotherapists. The overall aim with the workshops was to explore and identify co-care needs, important functionalities in an eHealth service to enable co-care, and its potential impact for PD care. In workshops 1-3, participants engaged in co-design to explore needs and generate ideas (see Figure 1). In the third workshop, the participants prioritized functionalities to include in a functional prototype of an eHealth service. The prototype was developed by a software developer in the time period between the third and fourth workshops (3 weeks), in collaboration with the workshop facilitators and researchers. Content to include in the prototype was collected from the participants. The PwP contributed by sharing their most recent medication list and prescriptions through an anonymous questionnaire, and HCPs contributed by sharing self-care instructions and assessment instruments that are used in routine care. The PwP user interface was designed for mobile devices (smartphone or tablet), and the HCP user interface was designed for a computer screen (see Multimedia Appendix 1). In the fourth workshop, the participants discussed their perceived usability and acceptance of the prototype and potential impacts. More details about the recruitment process, participants, and structure and content of the co-design workshops are described in [28]. The regional ethical committee approved the study (2015/2216-31/5).

#### Mini Fair for Parkinson’s Disease Research

After the workshop series, in October 2016, we demonstrated the co-care prototype at a Mini Fair for ongoing Parkinson’s disease research at Karolinska Institutet. After our demonstration, PwP and informal caregivers in the audience were invited to evaluate the prototype and its different functionalities in a questionnaire. The questionnaire was answered by 37 respondents: 31 PwP (14/31, 45% female) and 6 informal caregivers (3/6, 50% female). One of the responding PwP had also participated in the co-design workshops.

### Data Collection and Analysis

#### Co-Design

User needs and design ideas were collected in workshops 1-3 through note cards produced by the participants in co-design sessions, based on the nominal group technique [29], and focus group discussions with HCPs and PwP separately (workshop 3) [30]. In workshop 4, the prototype was evaluated in focus group discussions, first separately with HCPs and PwP and then collectively with all participants. All workshops were audio recorded.

We followed the principles of a qualitative thematic analysis to analyze the data in 6 phases, using both an inductive and deductive approach [31]. In the first phase, the handwritten note cards created by individual participants (n=139) and the ones created collectively (n=83) were transcribed and labeled with the co-design session number and participant role where applicable. Selected parts from the co-design sessions were transcribed to complement the note cards when more descriptive details were needed for the analysis. The focus group discussions were transcribed verbatim. In the second phase, we identified meaning units in the data and generated initial codes to reflect their content. In the third phase, the meaning units with their initial codes were printed out on individual paper slips and sorted deductively into themes guided by the research question: (1) experienced needs, (2) desired functionalities of a co-care service, and (3) expected value. Subthemes were created inductively by grouping data according to similarities and differences. We first categorized data from the co-design sessions and thereafter added data from the focus group discussions. In the fourth phase, we reviewed and discussed the thematization of all data. When agreement was reached between the authors, the thematic map (ie, themes, subthemes, codes, and meaning units) was transferred to mind-mapping software (FreeMind version 1.0.1). In the fifth phase, themes and subthemes were refined and renamed in several iterations until they reflected a condensed analysis of the participants’ expectations and desired eHealth functionalities to achieve co-care. In the final phase, we selected illustrative quotes that were translated from Swedish into English. Presented quotations are complemented with information about the source or respondent group (ie, note card, PwP or HCP) and workshop number (eg, WS1), which is provided in brackets.

#### Prototype Evaluation

The evaluation questionnaire contained 2 questions about general impressions and 7 questions about the perceived importance of different functionalities. The questions were answered using Likert-type response options with 5 levels. In 2 final questions, respondents were asked to list the 3 most important functionalities and were given the opportunity to provide their own suggestions. We analyzed the questions as Likert-type items, reporting variability as frequencies and percentage and central tendency as mode [32] (Multimedia Appendix 2).

## Results

### Co-Design

The qualitative analysis resulted in 2 main themes: (1) core eHealth functionalities and their expected values and (2) individual and organizational constraints.

#### Theme 1: Core eHealth Functionalities and Their Expected Values

**Figure 2:**
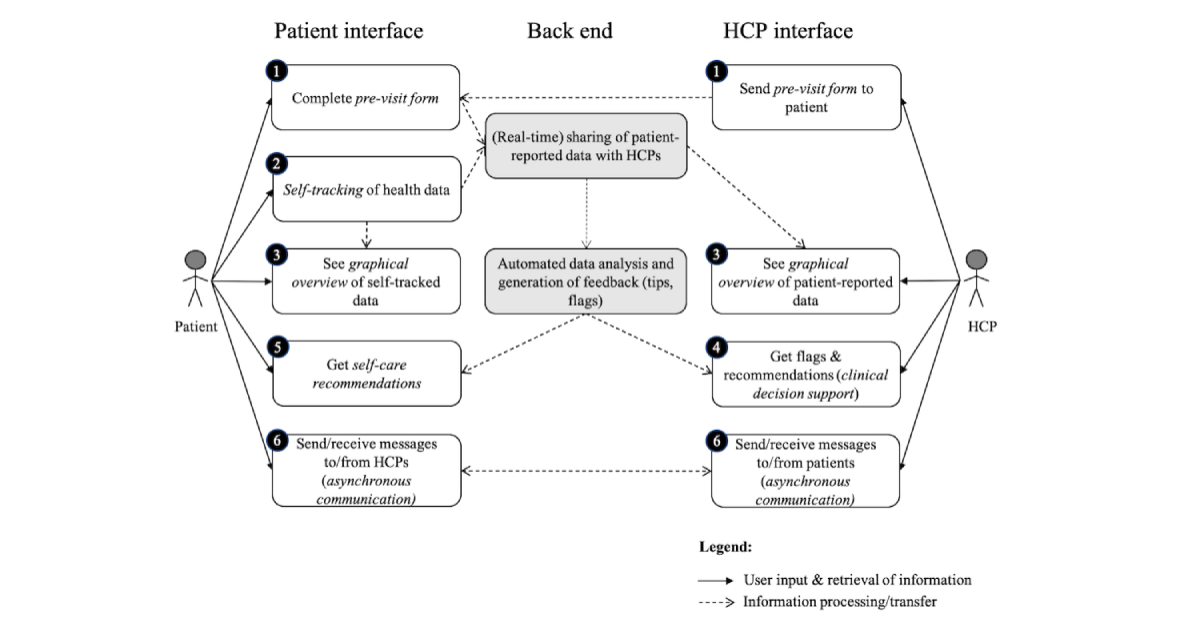
Use case diagram describing the desired functionalities of an eHealth service to support co-care in Parkinson’s disease care management, targeting the patient (left) and health care professionals (HCPs; right). The numbers in circles refer to the numbered eHealth functionalities in the text.

We identified 6 core eHealth functionalities for supporting PD co-care between PwP and HCPs. These include a previsit form (desired functionality 1), patient self-tracking (desired functionality 2), graphical overview (desired functionality 3), clinical decision support (desired functionality 4), self-care recommendation (desired functionality 5), and asynchronous communication (desired functionality 6) and are illustrated in a use case diagram (Figure 2) and described in the following text.

Regarding desired functionality 1, an electronic previsit form was suggested as a PwP-facing and HCP-facing functionality that enables both PwP and HCPs to prepare for planned patient visits and use time during visits more efficiently. The participants discussed that collecting information that is necessary for assessing the PwP’s health status during a visit can be a time-consuming activity. The dialogue in Textbox 1 illustrates how one of the physicians expressed their ambition and challenge of completing the Unified Parkinson’s Disease Rating Scale (UPDRS) during visits.

**Example of how one of the physicians expressed their ambition and challenge of completing the Unified Parkinson’s Disease Rating Scale (UPDRS) during visits.**

**Physician 1**: It is very challenging to find the time to complete it [UPDRS]. But when I do, I feel that I do – also for the long run – very high-quality work that is also easier to follow up.

[...]

**Facilitator**: Is this something the patient can do beforehand?

**Physician 3**: No, it's a status. Patients can help with other parts of it [UPDRS]. If there would be a validated translation in Swedish it could work.

**Physiotherapist**: Mhm, but there is none. [WS3]

The participants were of the opinion that electronic forms could be a way of collecting information from PwP prior to planned visits, which would be beneficial for both the PwP and HCPs. For example, main concerns and expectations for the upcoming visit, as well as a structured summary of experienced symptoms, medication intake, and diet could be reported beforehand. They pointed out that it would make work easier for HCPs and could support history taking and documentation:

Now patients come with their notes, which are not documented in any way [...] this may be a good instrument for documenting issues that may arise time and again.

HCP, WS4

One of the HCPs also pointed out that in this way, they would not need to remember to ask each of their patients about every possible issue on every occasion they meet, which would save time and enable more efficient consultations.

Regarding desired functionality 2, patient self-tracking was suggested as a PwP-facing functionality that allows PwP to track their own health and wellbeing on a daily basis. Self-tracking was discussed as a method that allows PwP to make their own measurements and register health-related parameters continuously whenever they experience a need for doing so. As one of the HCPs expressed, rather than limiting the collection of health data to the (few) instances when PwP have scheduled visits with HCPs, self-tracking could be “a way to collect [data] and monitor one's health condition over many years” [HCP, WS4]. The PwP believed that they would not mind spending time on self-tracking and filling in self-assessment forms if this could benefit their health. On the contrary, they saw value in the ability to monitor their health parameters over time. As one of the PwP described, this would make it “easier to understand what is Parkinson's disease and what is something else” [note card, WS2]. Also, they believed that they would feel more confident in their communication with HCPs because the self-tracked data would make it easier to adequately describe how their condition has varied over time.

The participants believed that HCPs would also benefit from PwP’s self-tracked data as the gathered information could make it easier to make adequate treatment decisions and provide insights about treatment adherence and effectiveness. If negative trends in health or wellbeing can be detected through self-tracking, they also anticipated an opportunity for more timely care interventions and prevention of undesired effects. Given these potential benefits, PwP expressed that the tracking and sharing of data would allow them to feel safer and calmer about their care.

Regarding desired functionality 3, the participants emphasized the need of a PwP-facing and HCP-facing graphical overview of health data collected through self-tracking and previsit forms. It should contain PwP’s reported symptoms and wellbeing, prescribed and consumed medication, as well as their tracked self-care activities, such as number of steps per day or other physical activities. As one of the PwP pointed out:

If you were to get out the best of this type of system, I think it would be to see trends.

PwP, WS4

By visualizing trends, the PwP expected to gain more understanding about their situation and thereby gain insights about the effectiveness of their self-care efforts.

The participants, mainly HCPs, further emphasized the need to integrate patient-reported data with other data sources, such as data documented in the electronic health record and the national Parkinson’s quality registry. As one of the HCPs pointed out:

But it is also convenient if everything is gathered in one and the same portal so that you don't need to find your way in different systems.

HCP, WS4

By gathering information from different sources in one overview, HCPs and PwP envisioned a strengthened collaboration also among HCPs*.* For example, a test result from the physiotherapist might be valuable for the physician when seeing the patient. Both HCPs and PwP also emphasized the possibility for improving collaboration with primary care:

And that possibly also primary care could get access to some of this, maybe not to use it, but to look into the system.

PwP, WS4

Regarding desired functionality 4, the need for HCP-facing clinical decision support functionality was emphasized by the HCPs. In particular, they desired automated guidance for planning when to schedule patient visits based on PwP’s individual needs:

The system signals when it's time for a visit or a telephone contact. [...] If there are several issues, we may need to schedule an earlier consultation.

HCP, WS4

Automated flags and alerts were suggested as a type of decision support functionality that could support HCPs in planning consultations according to identified needs, rather than a routine care protocol that does not consider the health status of individual patients. For example, the HCPs desired to be notified through alerts or flags that indicate the occurrence of extraordinary events or negative trends based on patient-reported data. As one of them reflected:

When something does not follow the pattern, that you react earlier, and then it may be easier to manage; not wait until the next follow-up visit which may be months ahead, and then it turns into a big problem instead.

HCP, WS4

The participants emphasized that automated alerts and recommendations based on patient-reported data are necessary for HCPs to make individual adjustments in care plans as it would not be possible for them to actively monitor all the patients’ self-tracking data. As one of the HCPs pointed out:

It [the anticipated eHealth system] needs to be so good that it simplifies - and not just by providing the doctor with information. It needs to be processed.

HCP, WS1

Regarding desired functionality 5, provision of self-care recommendations was one of the essential PwP-facing functionalities that the participants emphasized. As one of the PwP described it:

Self-care is something we do every day. All of us who have Parkinson's disease [...] It's when we get uncertain about our self-care that we need this - to look something up. It can be about symptoms, general wellbeing… some uncertainty we cannot manage on our own. That's when we need this.

PwP, WS1

The PwP expressed a desire for information about the causes and determinants of PD, symptoms, treatment, and ongoing research. Moreover, to be able to manage their own health with more confidence, PwP expressed that they need general as well as individually tailored recommendations for self-care. In particular, they desired recommendations regarding administration of medication, diet, physical activity, and exercises they could perform on a daily basis (eg, “What and how should I exercise?” or “What can I do to feel better?” [note cards, WS3]).

With adequate support, the participants expected that PwP could take responsibility for more of the activities important for their health and thus be more autonomous in their self-care*.* As one of the participants expressed:

It is possible to support patients in different ways – if there is something to, do they get more independent, I think.

HCP, WS4

One of the HCPs suggested that a sophisticated eHealth service would ideally generate simple self-care advice based on patient-reported data:

I imagine that a form of self-care could be that you feed in a bunch of data and then something pops out of the eHealth service. But I also understand that it is difficult to do. [...] But simple things: “Have you tried to take the medication without food?” or, if feeling nauseous: “Have you tried to take it with food?”

HCP, WS1

The possibility of a self-care scoring system and rewards was discussed as a means to motivate PwP to engage in their self-care. However, the idea of rewards and who should receive these was controversial. As one of the PwP emphasized:

Maybe health care should receive the movie theatre gift card instead [of patients], so that they log into the system – because I think that's where the resistance will be.

PwP, WS4

However, as one of the HCPs pointed out:

I think that can be intimidating for our staff. We are so pressured. If there is too much of that [rewards and bonus points], I think there will probably be many who choose not to use it [the system].

HCP, WS4

Regarding desired functionality 6, text-based messaging for asynchronous communication was suggested as PwP-facing and HCP-facing functionality. The PwP emphasized the need for continuous and maybe more frequent and regular contact with their HCPs. The participants discussed that a messaging service would make it easier for PwP to get in contact with their HCPs, for example to inform about newly experienced symptoms or to ask for renewals of prescriptions or health certificates — issues that may be resolved without having to meet face-to-face. However, the participants maintained that the idea is not to replace physical visits. As one of the PwP pointed out:

I just think that the planned visit with the health care professional is very important as a check-up — so that this is not systematized too much without noticing when the system goes to hell. The concept is good, but we need — the ultimate checkpoint is to see the patient in front of the health care professional.

PwP, WS1

The main expected benefit of asynchronous communication was better access to care and more timely support because PwP would be able to contact and reach health care at any time when a need occurs. As one PwP expressed:

This is a way to break into health care.

PwP, WS4

As one of the HCPs described:

I also think that, many patients we meet want to get in contact, it is difficult to call and no one responds, so it can be very convenient and calming to feel that “I have reported an issue; I have sent it and received a confirmation that it is sent to [HCP].”

HCP, WS4

#### Theme 2: Individual and Organizational Constraints

Two subthemes of constraints were identified that need to be addressed for succeeding with an eHealth service for co-care. These reflected constraints on an individual level (eg, relating to eHealth literacy and technology acceptance) and constraints on an organizational level (eg, how teamwork and collaboration between PwP and HCPs are organized).

Regarding the subtheme of eHealth literacy and acceptance, the necessity of eHealth literacy and acceptance was discussed as a major constraint of eHealth services. The participants pointed out that communicating through an eHealth service may not improve access to care for all PwP as some individuals may not be willing or able to use an eHealth service. As one of the HCPs pointed out:

Everyone will not be able to manage this and then we come to what [physician] said — that we need flexibility. There are those who will never have the energy to acquire the knowledge that is needed to manage this [anticipated eHealth system].

HCP, WS1

Specifically, motor symptoms of PD may cause difficulty filling in forms. Concerns were also raised in relation to nonmotor symptoms:

Unfortunately, one of the nonmotor symptoms is that you don't have the energy to fill in these forms. Parkinson-related fatigue is a concern.

PwP, WS4

An HCP also raised that some health problems may require informal caregivers’ collaboration:

In case of hallucinosis or impulse control problems, patients won’t report these issues themselves. In such cases, family members would need to report.

HCP, WS4

Regarding the subtheme of teamwork and administrative workload, the participants anticipated that an eHealth service for co-care may require HCPs to engage more in teamwork.

But they [health care] need to somehow organize themselves. This [anticipated eHealth service] could maybe enforce a more holistic perspective.

PwP, WS4

They emphasized the importance of organizing the team around the patient and clarifying roles and responsibilities:

It needs to be limited, so that the patient-reported issues do not end up being owned by many but addressed by no one.

HCP, WS4)

For example, they stressed that it is important to know who should take responsibility for corresponding with the patient.

The participants further raised that an eHealth service for patient-provider collaboration might cause additional administrative workload for HCPs and thereby limit the time available to interact face-to-face with patients. As one HCP expressed it:

My fear is that one gets tied up with the system and forgets about what is important, the patients.

HCP, WS4

As one of the PwP expressed:

If a nurse or physician communicates something to a patient, does this have to be documented in the patient’s record or not? This is something health care will come down on immediately. And if it needs to be documented, this implies a double documentation burden for the HCP.

PwP, WS4

HCPs also feared that there is a risk that individual patients might overuse the opportunity to report health issues, which would increase HCPs’ workload. Hence, this could scare HCPs from using this type of eHealth service. As one of the HCPs pointed out:

Because it is painful [...] not to be able to meet existing expectations. And there will be conflicts in the sense that we know that we would be able to do things better, but we can’t.

HCP, WS1

### Prototype Evaluation

The majority of the 37 questionnaire respondents (31/37, 84%) perceived that they would benefit from an eHealth service similar to the demonstrated prototype, while some (5/37, 14%) were neutral and one (1/37, 3%) saw no benefit. Using rewards was perceived as a benefit by 24 (24/37, 65%), while 12 (12/37, 32%) were neutral and one (1/37, 3%) saw no benefit. All prototype functionalities were rated as very important or important by the majority of respondents (ranging from 86% to 97% per functionality). Previsit forms were rated lowest with a mode of 4 (Important). For all other functionalities (self-tracking, graphical overview, self-care recommendations, asynchronous communication), the mode was 5 (Very important). The 3 functionalities that were rated most important were the ability to *send* messages to HCPs (asynchronous communication), graphical overview, and self-tracking. The ability to *receive* messages from HCPs got the fewest ratings as one of the 3 most important functionalities, followed by the previsit forms. The following additional functionalities were suggested: accumulated statistics, synchronization with new models of care, drug information and interaction alerts, and reminders to take medication. Details are presented in Multimedia Appendix 2.

## Discussion

### Principal Findings

The aim of this study was to explore how co-care could be operationalized in PD care, supported by eHealth. We identified 6 core eHealth functionalities that were desired by both PwP and HCPs, and all of them were rated important or very important in a group of PwP and informal caregivers that did not participate in the co-design. The co-design participants believed that these functionalities could contribute to higher quality of care in terms of safety, timeliness, effectiveness, efficiency, and patient-centeredness. Concerns that were raised included constraints on individual and organizational levels, which would need to be addressed to succeed with the implementation of a future co-care service.

### Comparison With Previous Work

In the comparison of our results with prior work, we categorized the 6 desired functionalities into 3 groups, based on their purpose: (1) collection and sharing of health data (previsit forms, self-tracking), (2) feedback and recommendations (graphical overview, self-care recommendations, clinical decision support), and (3) asynchronous communication.

#### Functionalities for Collection and Sharing of Health Data

Self-tracking was described as functionality that is initiated and driven by PwP. In line with the participants’ expectations, previous research has reported that self-tracking could contribute to a deeper understanding about PD manifestations among PwP and enhance both self-care and communication with health care, while also pointing out the importance for PwP to find a balance between the burdens and benefits of self-tracking [33]. The PwP in our study were not worried about the burden of frequent tracking, but the high attrition rate of eHealth services in general needs to be considered [34]. In contrast to self-tracking, previsit forms were suggested as a type of data collection that could be initiated by HCPs to save time during consultations. Previous research indicates that self-reported symptom assessments based on the UPDRS may be a viable option as patients’ own assessments are not less reliable than clinicians’ assessments [35]. Other patient-oriented assessment methods still need refinement (eg, the assessment of visual hallucinations, which are common among PwP [36], or assessments of cognitive impairments [37]). Our study participants emphasized the importance of including open questions in previsit forms that allow PwP to describe their main concerns in their own words. However, previous research has shown that discrepancy in patients’ and HCP’s perceptions of which information is important for health care staff to know and respond to may lead to disappointments [38]. A central aspect in the design of such functionalities is the “alignment of concerns” between patients and HCPs to ensure that the shared information is considered meaningful, actionable, and feasible from the perspectives of both patients and HCPs [39].

#### Functionalities for Providing Feedback and Recommendations

Feedback can support PwP in their self-care by providing insights on aggregated symptoms and medication data [40]. While there is an upsurge of self-monitoring applications available to support PD care, comprehensive systems that can support both the assessment of health data and provide treatment recommendations are limited [15,22]. A recent study identified that available eHealth solutions for PD do not always present graphical visualizations of self-tracked data to patients, which defeats the purpose of supporting individuals’ self-care [24]. Research about clinical decision support in PD focuses largely on early detection and diagnosis of PD [41,42], but the participants in our study stressed the value of clinical decision support and self-care recommendations related to the management of already diagnosed PD. Active feedback, including alerts and personalized recommendations, was considered essential to support individual needs-based health care and self-care. In relation to rewards, previous research corroborates our study participants’ mixed feelings about game-based approaches to enhance motivation and suggests that alternative motivation strategies should be considered [43]. For HCP interfaces, the importance of workflow integration to support data-driven consultations has been emphasized [44]. A European Union–funded project has reported on the design of a clinical decision support system for PD that takes a holistic approach, which is in line with the functionalities suggested in this study [45-47]. The researchers emphasize the importance of enabling shared decision making [46]. Machine learning techniques and medical knowledge are used to generate alerts and suggest appropriate actions to patients, informal caregivers, and health care professionals [47].

#### Functionalities for Asynchronous Communication

To add flexibility in patient-provider collaboration and improve access to care, the participants in this study emphasized the need for functionality to support 2-way asynchronous communication between PwP and HCPs. Limitations in access to PD care in Europe are characterized by a lack of consultations with PD specialists [48], a need for more multidisciplinary care [49], and PD consultations that are based on clinicians’ routines rather than driven by patient needs [50]. If eHealth is used effectively, it has been suggested that the traditional model of care with annual follow-up visits may in future be decomposed into several shorter needs-based consultations [51], which our study participants also expected. However, it has been reported that eHealth services for PD still have a tendency to prioritize the doctor’s perspective, while patients’ and informal caregivers’ needs may not be fully supported [24]. For example, PwP are rarely able to initiate a consultation with their HCPs or signal that their treatment needs adjustment [24]. The PwP in our study emphasized the importance of being able to contact HCPs with free-text messages allowing them to ask questions or signal experienced needs or concerns. However, based on a Cochrane review, there is yet little evidence to justify the use of text messages to support self-management [52]. Therefore, despite the anticipated value of functionality for asynchronous communication that can be initiated by PwP, the potential risks and benefits need further research.

#### Addressing Individual and Organizational Constraints of Using eHealth for Co-Care

As has been shown in previous research, poor eHealth literacy and acceptance could lead to disparities [53]. In particular, factors such as age and disabilities have been negatively associated with the digital divide [54]. The median age of PD onset is 60 years [2]. In comparison, the median age of the PwP in our study was 73 years. To address symptom-related disabilities of PD, design guidelines for touch screen gestures have been suggested [55], as well as calibration of touch screen sensitivity [56]. Previous research also emphasizes the important role of informal caregivers in supporting self-care and symptom assessments [57,58]. Acknowledging caregivers’ role in self-care, it has been suggested that eHealth services for PD should be designed to support collaboration between PwP and their informal caregivers [59]. While our study participants also regretted the absence of informal caregivers in the co-design process, they nevertheless expected that the main challenge ahead would be to engage HCPs. We acknowledge that the implementation and adoption of PD technologies in health care require integration with clinical workflow [19].

### Limitations

A limitation of this study is that the prototyped eHealth service has not been implemented and evaluated in clinical practice. Thus, our study does not allow us to draw conclusions about the actual value of the desired eHealth functionalities for co-care, including cost-effectiveness and clinical outcomes. Nevertheless, we believe that the multistakeholder co-design workshops provided an effective forum for the PwP and HCPs to discuss and align their concerns, which has been described as a prerequisite for successful design and implementation [39]. This may have been confirmed by the high ratings of the importance of all co-designed functionalities in our evaluation questionnaire. However, these results should be interpreted with caution as the questionnaire was not based on a validated instrument. Our intention was to use the results to guide the next step in the design process, rather than as a summative evaluation of the prototype.

The transferability of our results is inevitably limited by contextual factors of the overall study design, the participant constellation, and the Swedish health care system and standards of care. As has already been discussed elsewhere [28], the constellation of participants had several shortcomings: We failed in our attempt to involve informal caregivers; most of the participating PwP were highly educated and experienced (ie, expert patients); and there were existing professional or patient-provider relationships between some of the participants. This may have influenced the results to not fully reflect the needs of all stakeholder groups. Because the participants were recruited from different health care organizations, a contextual analysis of (local) care workflows was not considered meaningful at this stage. Instead, we discussed care practices based on the national guidelines for PD care in Sweden [60]. Despite these limitations, we believe that our results capture experienced needs and desired eHealth functionalities for co-care that may be of relevance also in other PD settings, as well as other areas of chronic care management.

### Conclusions

This study adds to our knowledge on how co-care in PD care could be operationalized. It provides a description of 6 core eHealth functionalities that were desired by PwP and HCPs to support co-care. Co-care implies a shift from episodic routine-driven care to more flexible care management that is driven by the mutual needs of patients and HCPs and supported by active information exchange between them, as well as automated information processing to generate patient-specific advice. While various eHealth applications have been developed and tested for different purposes, as of yet, we lack evidence for services that enable PD co-care by supporting the mutual needs and requirements of PwP, informal caregivers, and HCPs. More research is needed to further explore the concept and operationalization of co-care in chronic care management and what it means for self-care, health care, and ultimately, individuals’ health and wellbeing.

## Appendix

Multimedia Appendix 1 Screenshots of the co-care prototype that was developed.

Multimedia Appendix 2 Descriptive statistics of the evaluation questionnaire.

## Abbreviations

HCP: health care professional
PD: Parkinson's disease
PwP: person/people with Parkinson’s disease
UPDRS: Unified Parkinson's Disease Rating Scale
WS: workshop
