# Supplementary material for: How People with Parkinson's Disease and Health Care Professionals Wish to Partner in Care Using eHealth: Co-Design Study
Source: J Med Internet Res. 2020 Sep 21;22(9):e19195. doi: 10.2196/19195 (PMC7536604; doi:10.2196/19195)
Supplement: Multimedia Appendix 1 [file jmir_v22i9e19195_app1.pptx]

## Slide 1
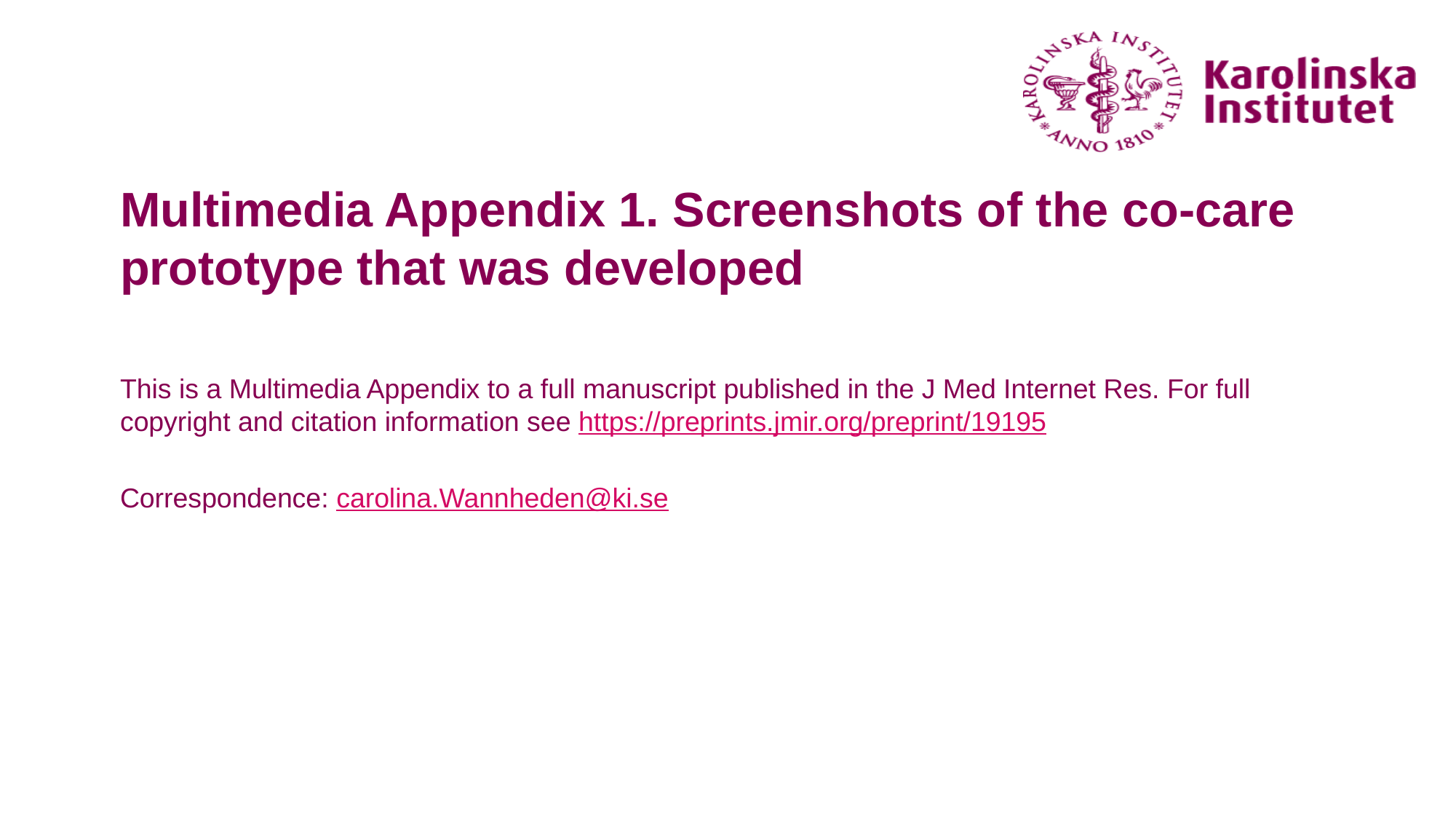

# Multimedia Appendix 1. Screenshots of the co-care prototype that was developed
This is a Multimedia Appendix to a full manuscript published in the J Med Internet Res. For full copyright and citation information see https://preprints.jmir.org/preprint/19195
Correspondence: carolina.Wannheden@ki.se

## Slide 2
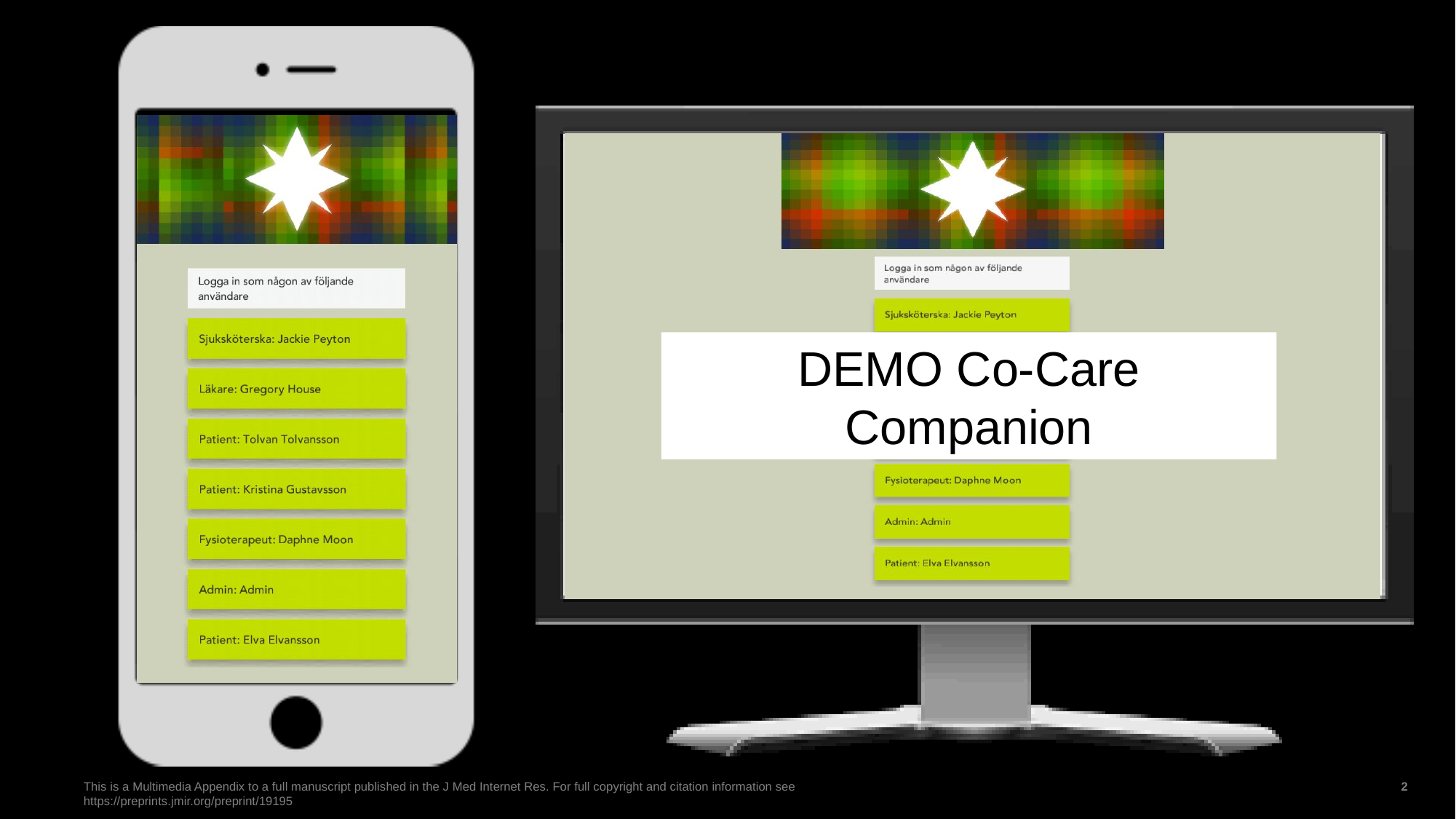

DEMO Co-Care Companion
This is a Multimedia Appendix to a full manuscript published in the J Med Internet Res. For full copyright and citation information see https://preprints.jmir.org/preprint/19195
2

## Slide 3
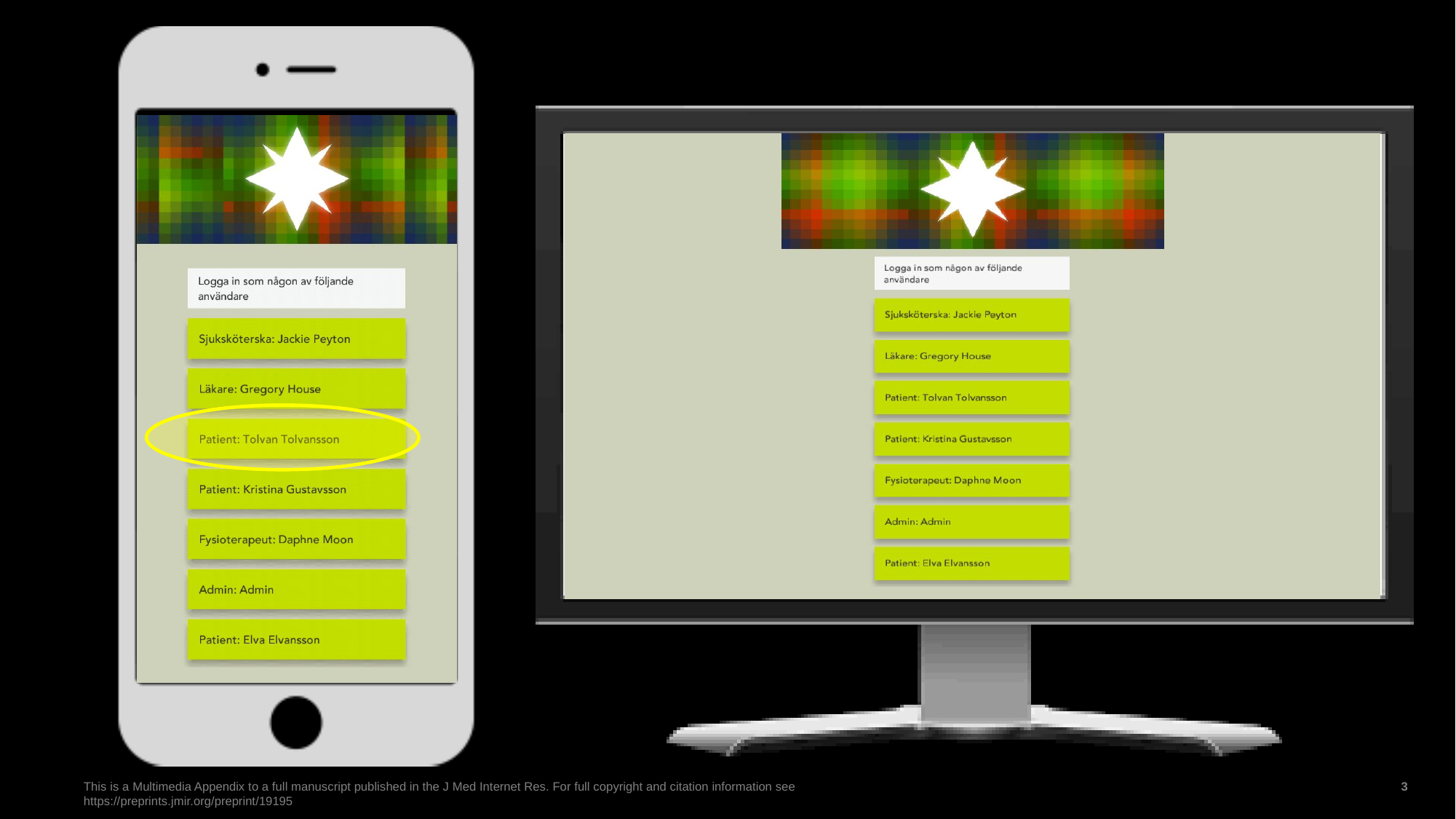

This is a Multimedia Appendix to a full manuscript published in the J Med Internet Res. For full copyright and citation information see https://preprints.jmir.org/preprint/19195
3

## Slide 4
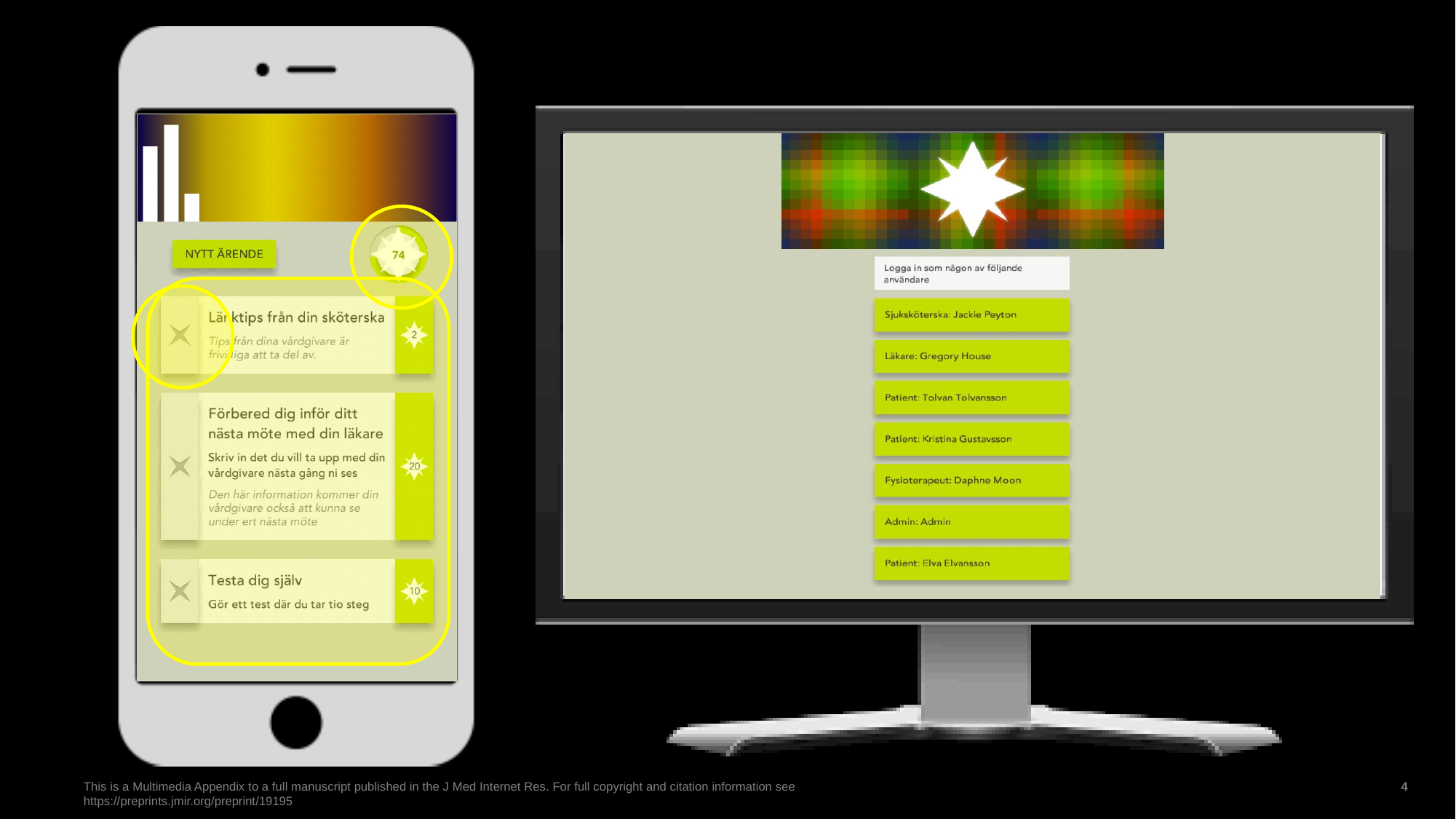

This is a Multimedia Appendix to a full manuscript published in the J Med Internet Res. For full copyright and citation information see https://preprints.jmir.org/preprint/19195
4

## Slide 5
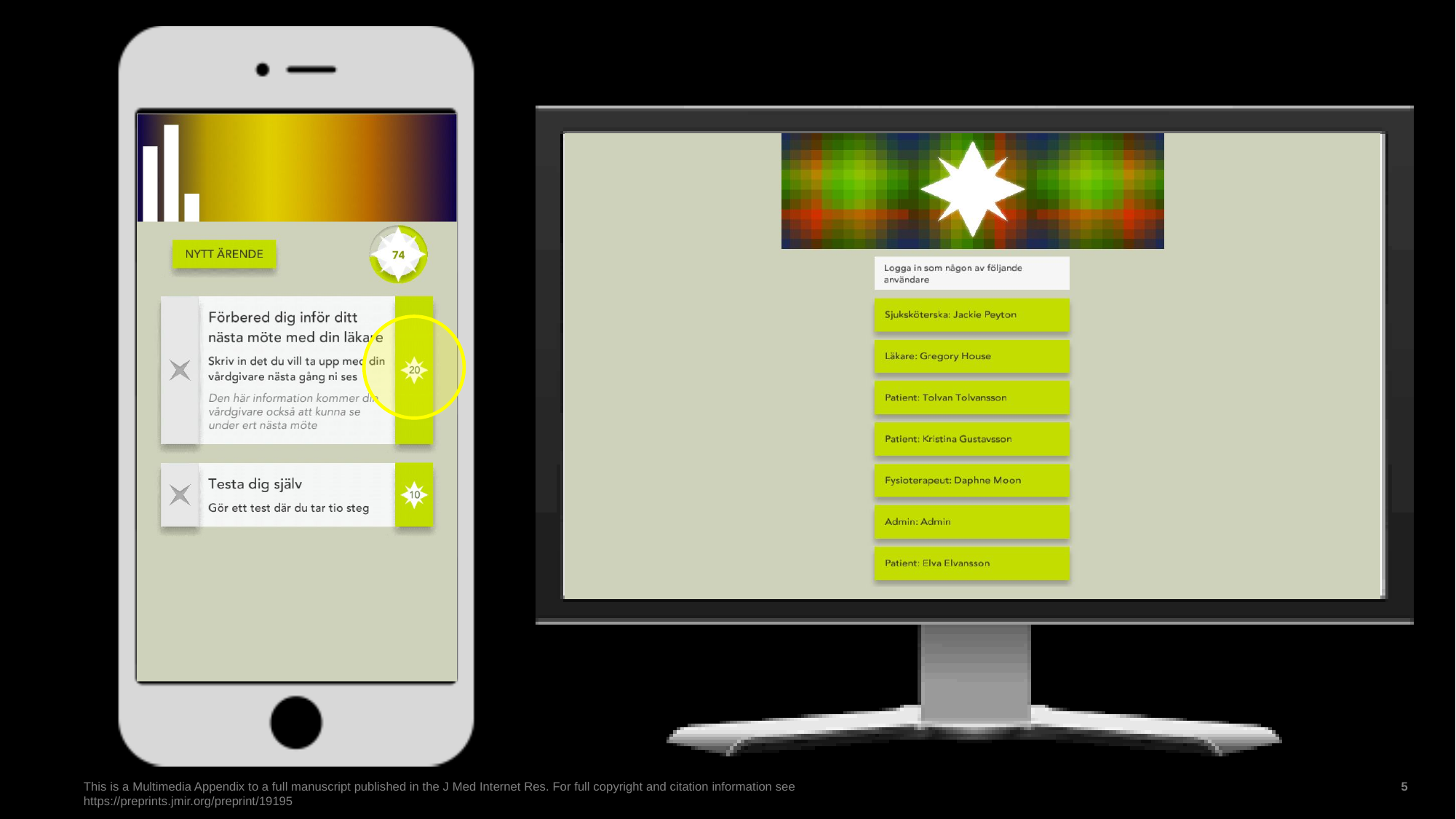

This is a Multimedia Appendix to a full manuscript published in the J Med Internet Res. For full copyright and citation information see https://preprints.jmir.org/preprint/19195
5

## Slide 6
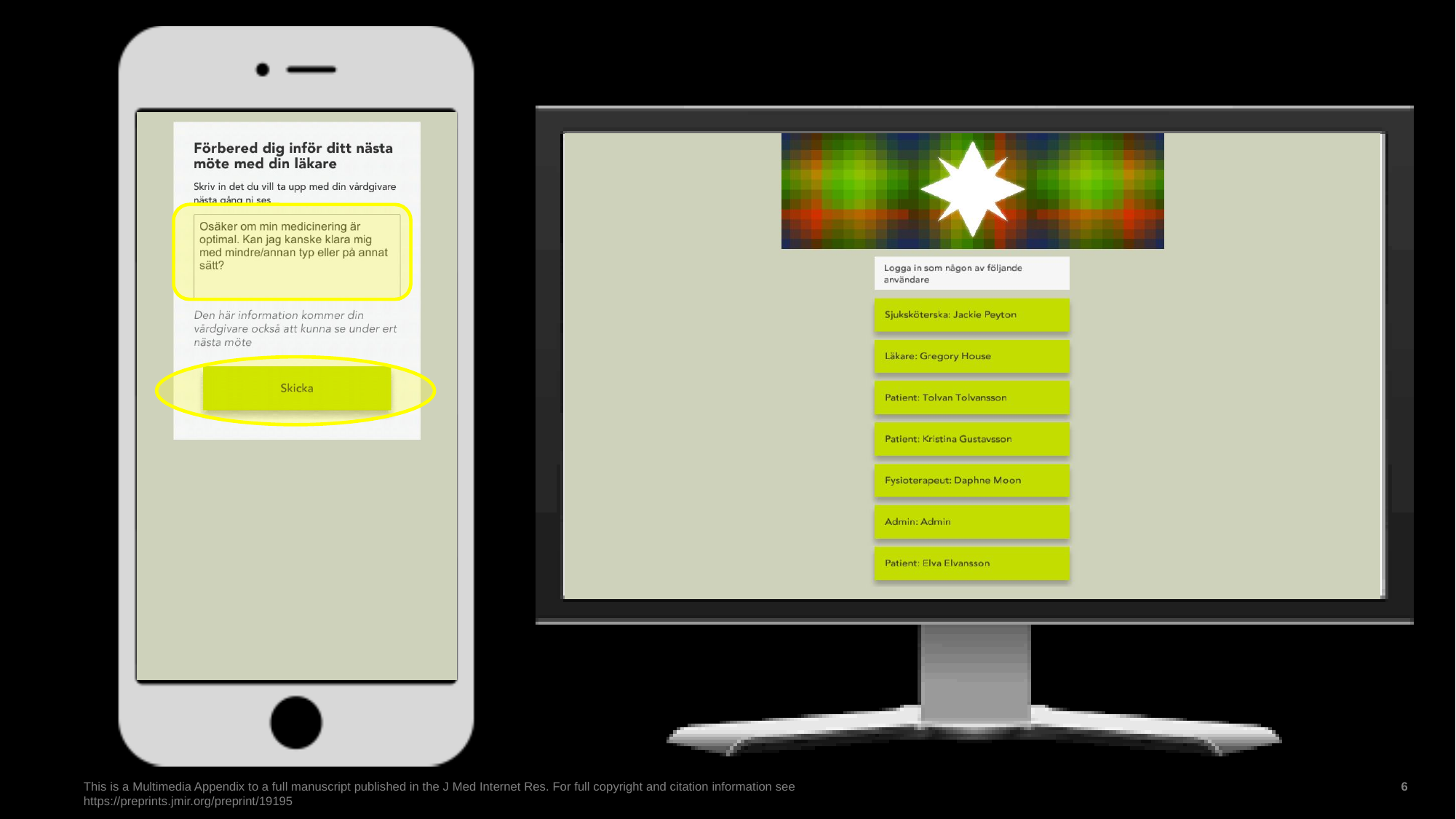

This is a Multimedia Appendix to a full manuscript published in the J Med Internet Res. For full copyright and citation information see https://preprints.jmir.org/preprint/19195
6

## Slide 7
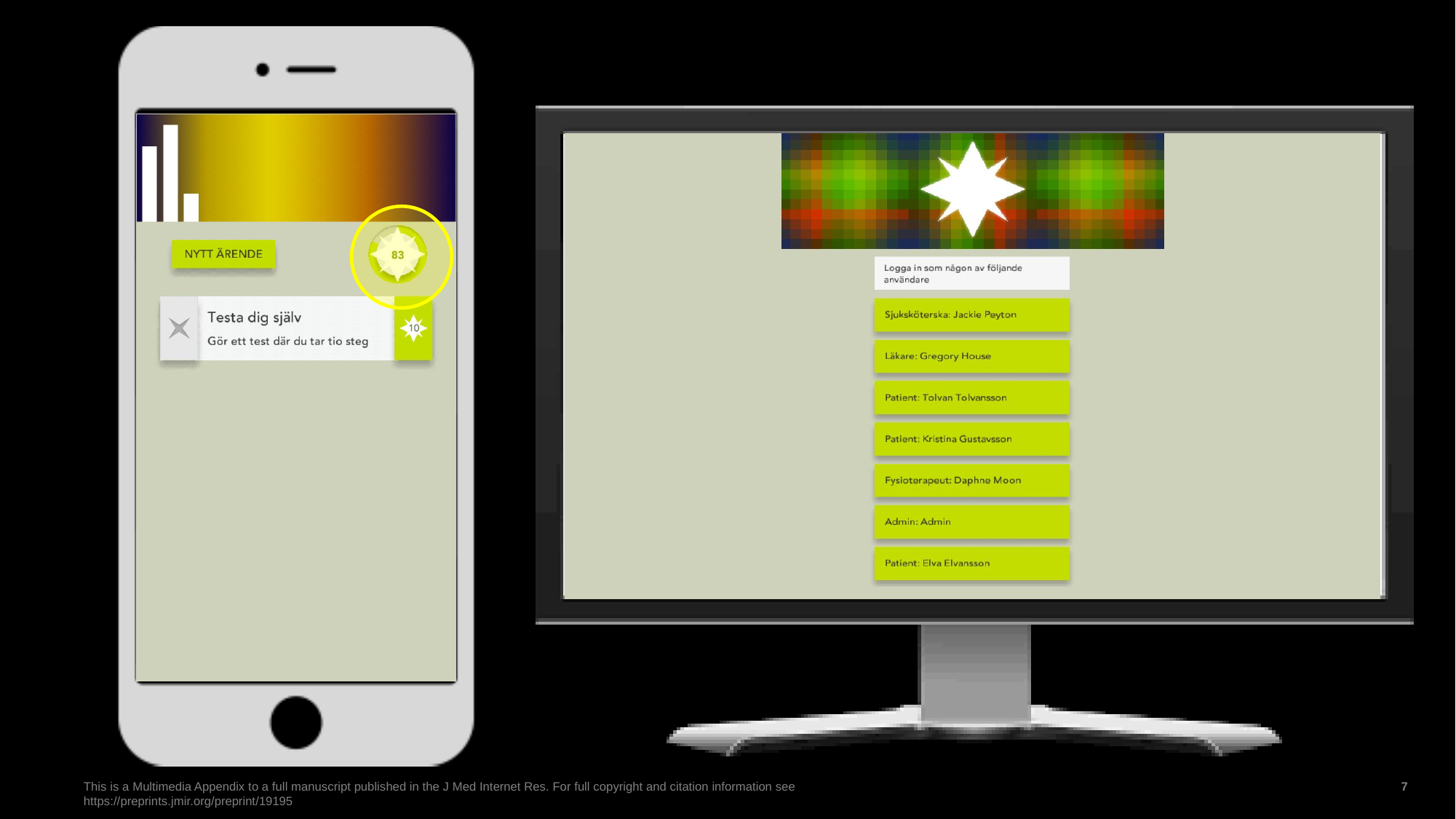

This is a Multimedia Appendix to a full manuscript published in the J Med Internet Res. For full copyright and citation information see https://preprints.jmir.org/preprint/19195
7

## Slide 8
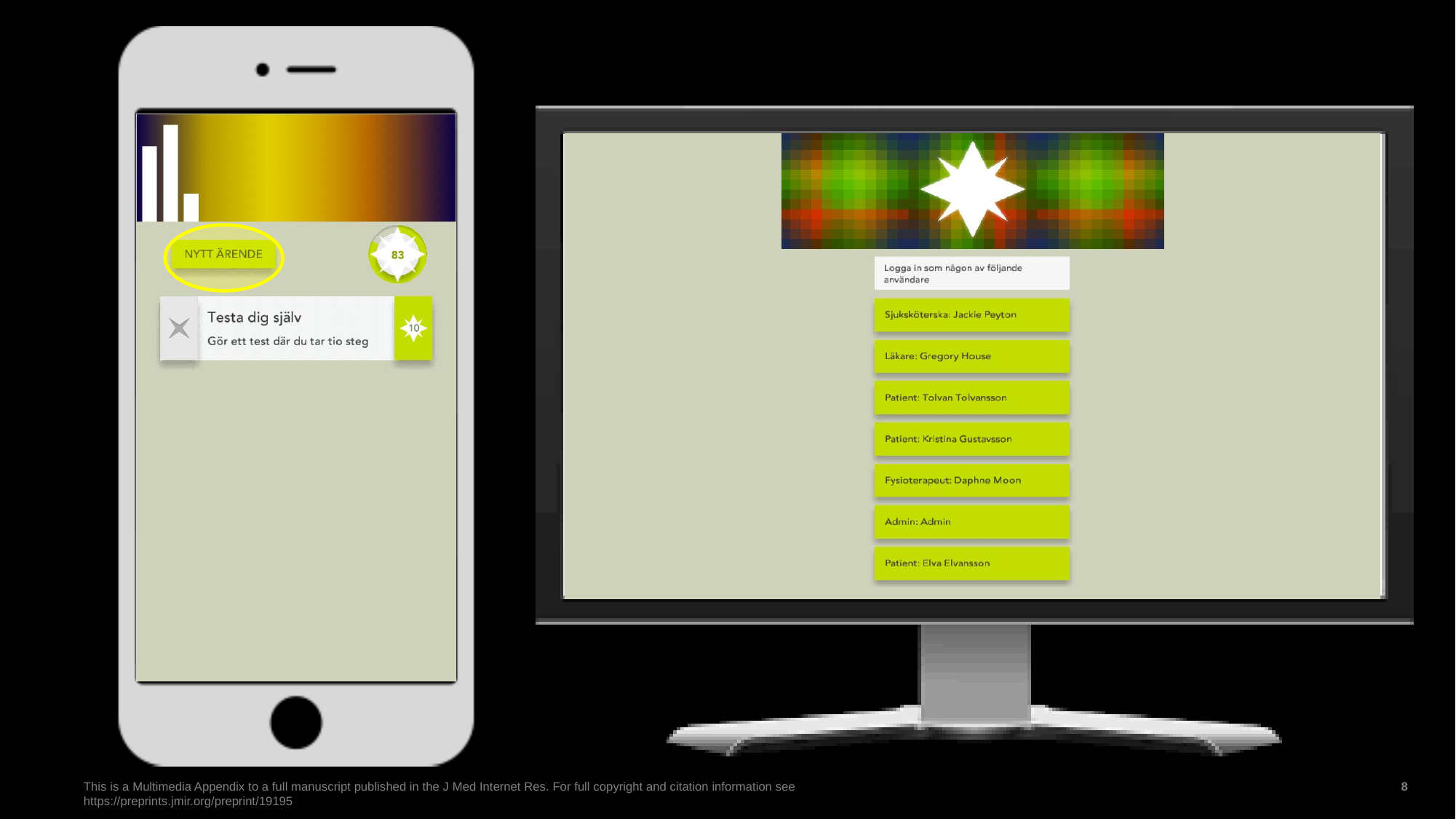

This is a Multimedia Appendix to a full manuscript published in the J Med Internet Res. For full copyright and citation information see https://preprints.jmir.org/preprint/19195
8

## Slide 9
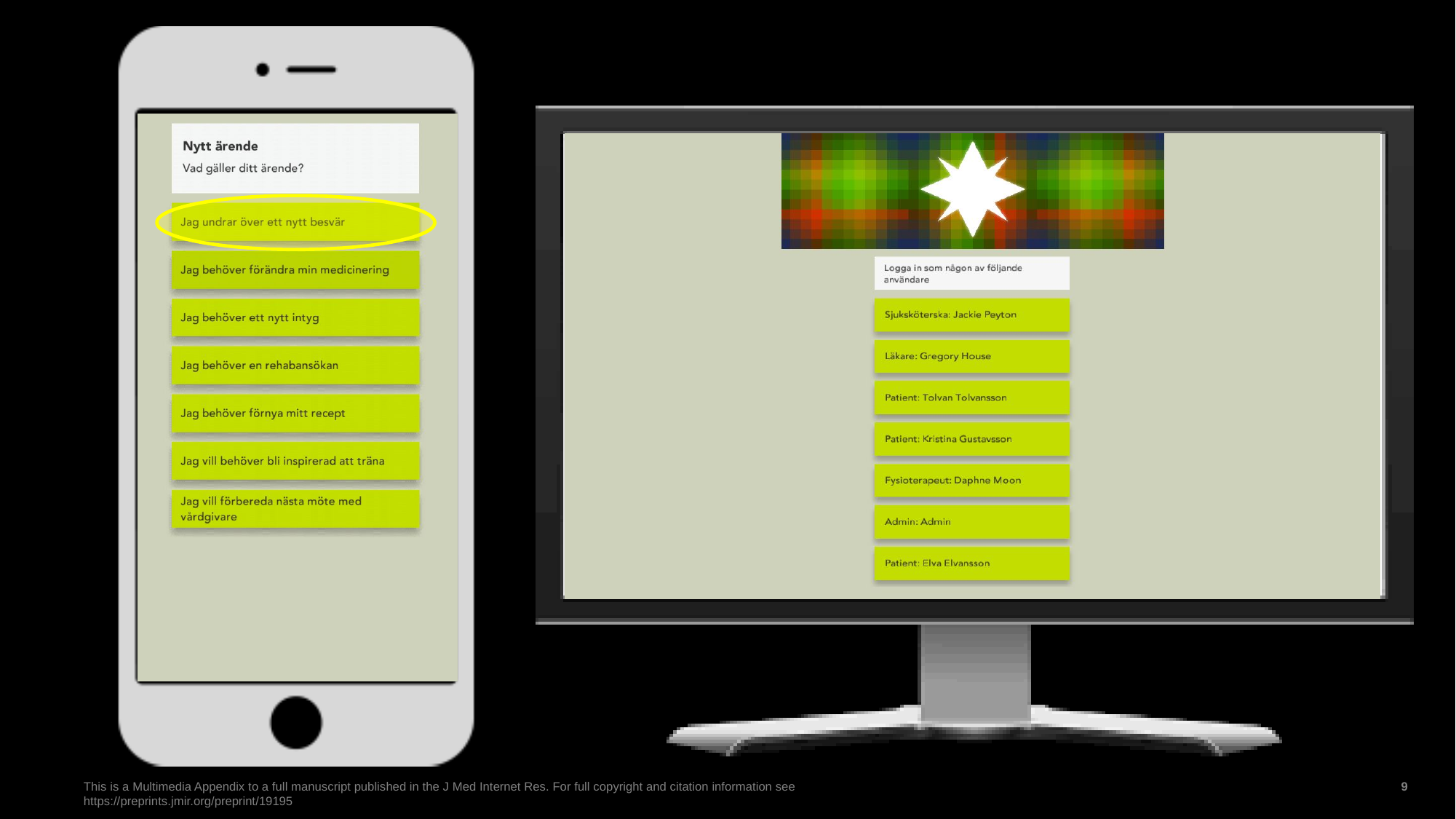

This is a Multimedia Appendix to a full manuscript published in the J Med Internet Res. For full copyright and citation information see https://preprints.jmir.org/preprint/19195
9

## Slide 10
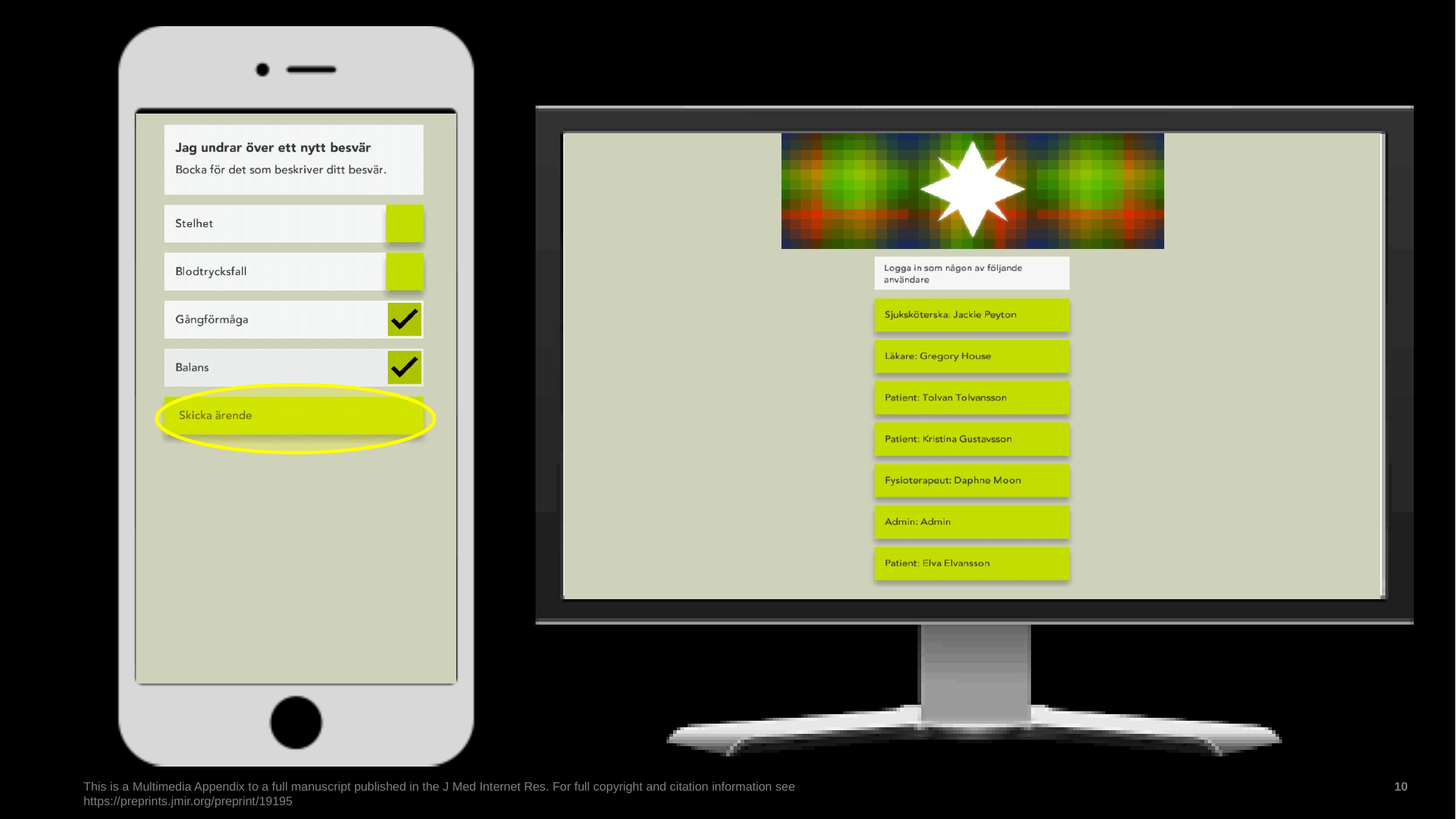

This is a Multimedia Appendix to a full manuscript published in the J Med Internet Res. For full copyright and citation information see https://preprints.jmir.org/preprint/19195
10

## Slide 11
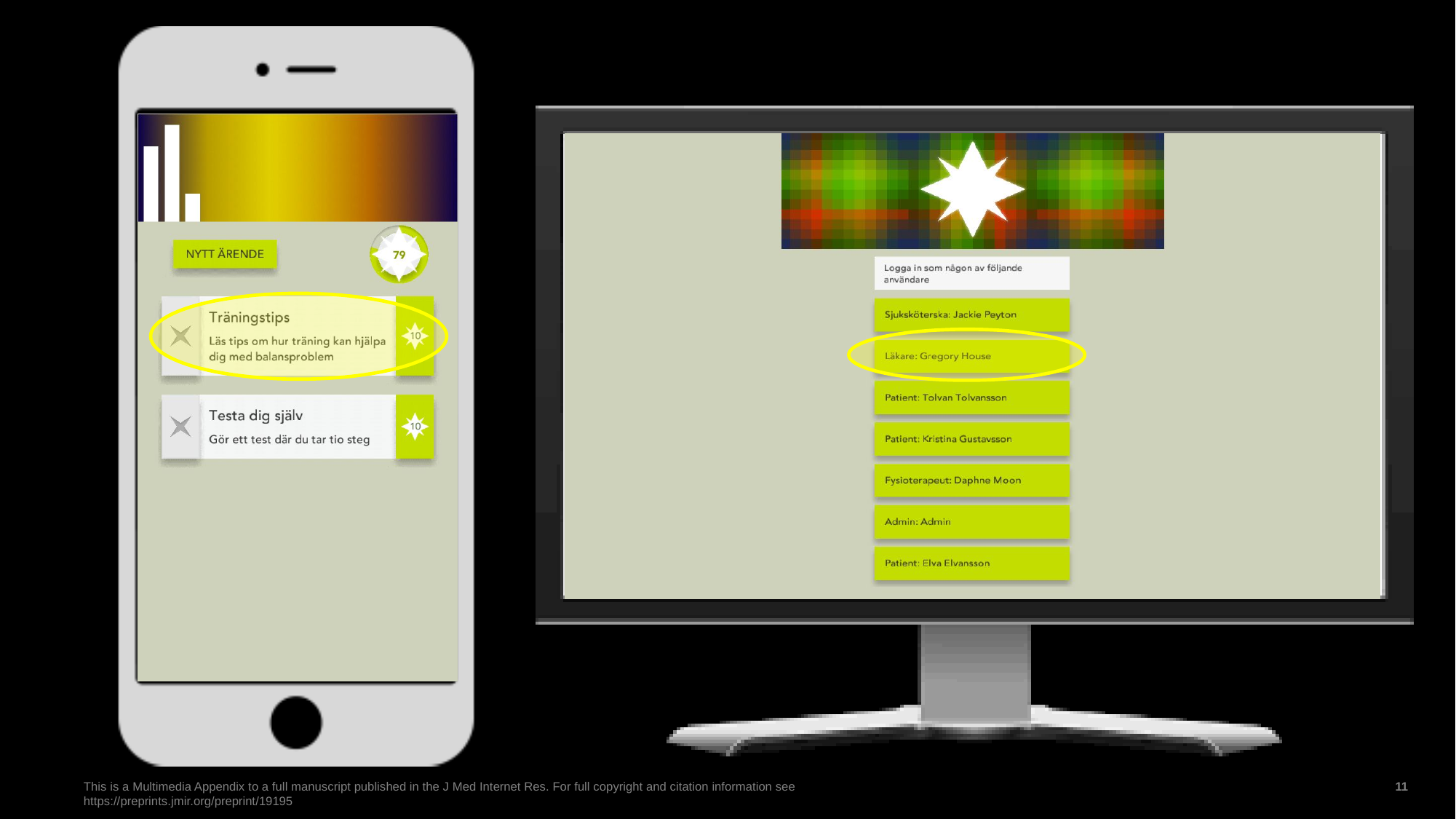

This is a Multimedia Appendix to a full manuscript published in the J Med Internet Res. For full copyright and citation information see https://preprints.jmir.org/preprint/19195
11

## Slide 12
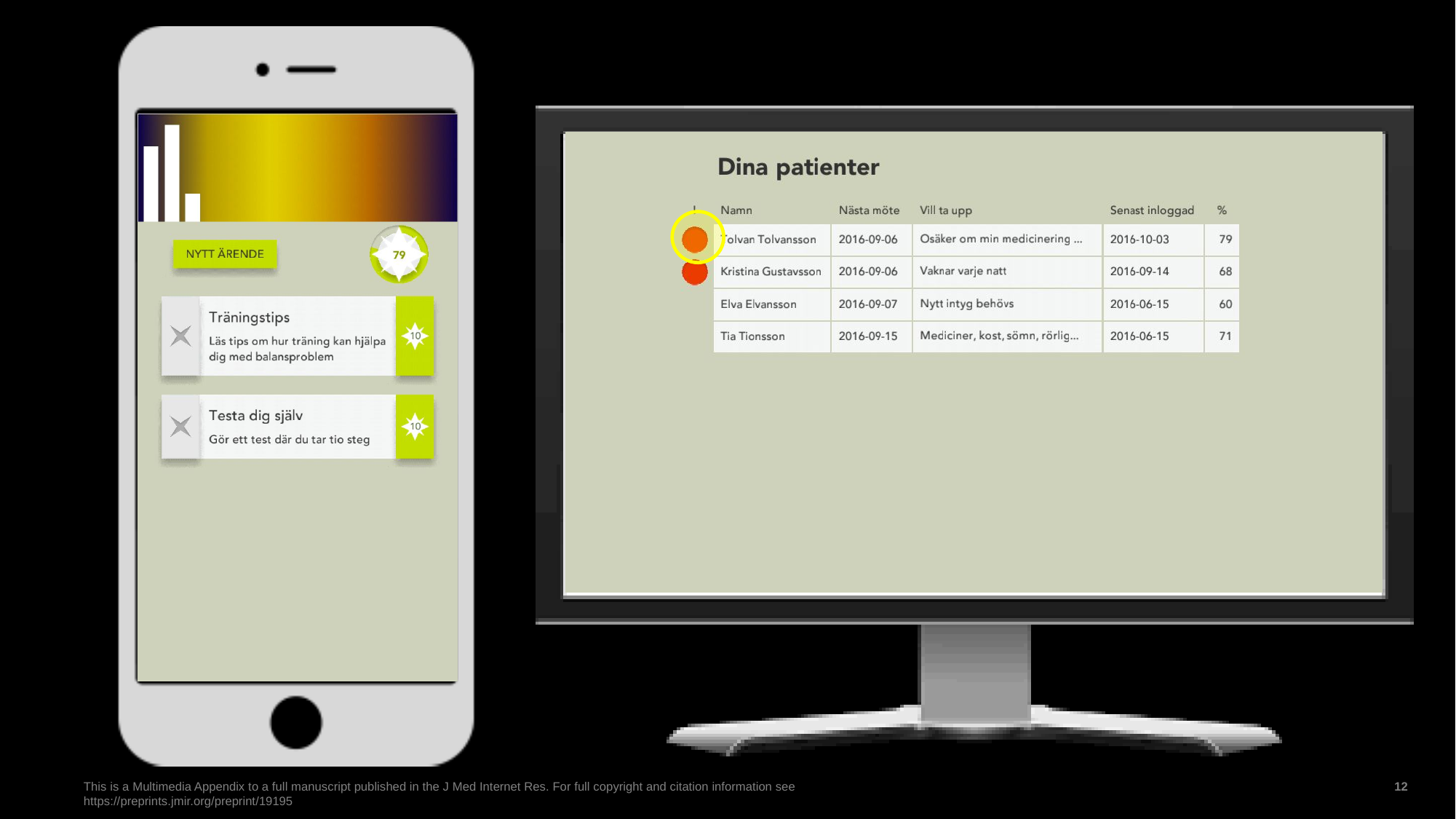

This is a Multimedia Appendix to a full manuscript published in the J Med Internet Res. For full copyright and citation information see https://preprints.jmir.org/preprint/19195
12

## Slide 13
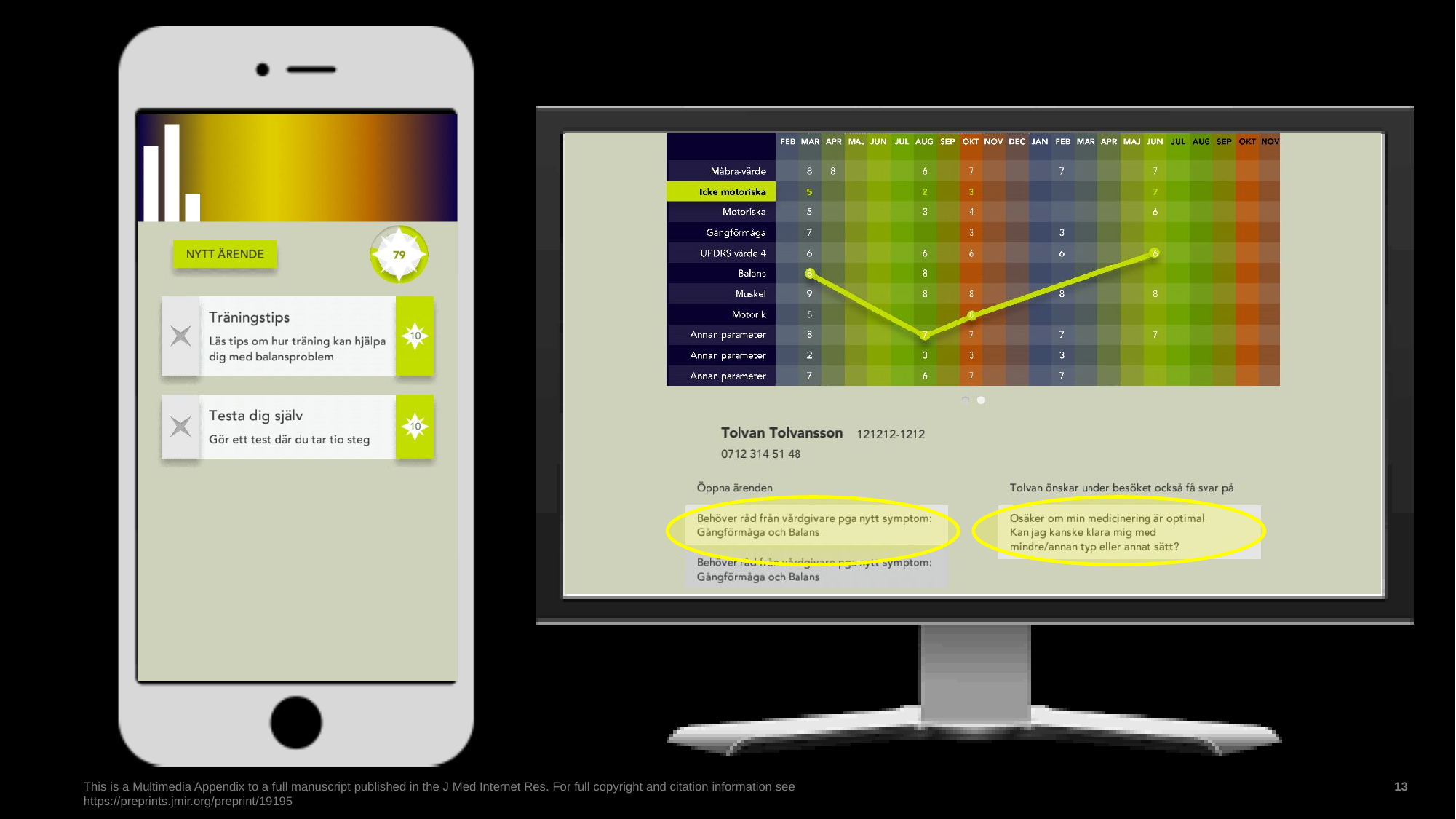

This is a Multimedia Appendix to a full manuscript published in the J Med Internet Res. For full copyright and citation information see https://preprints.jmir.org/preprint/19195
13

## Slide 14
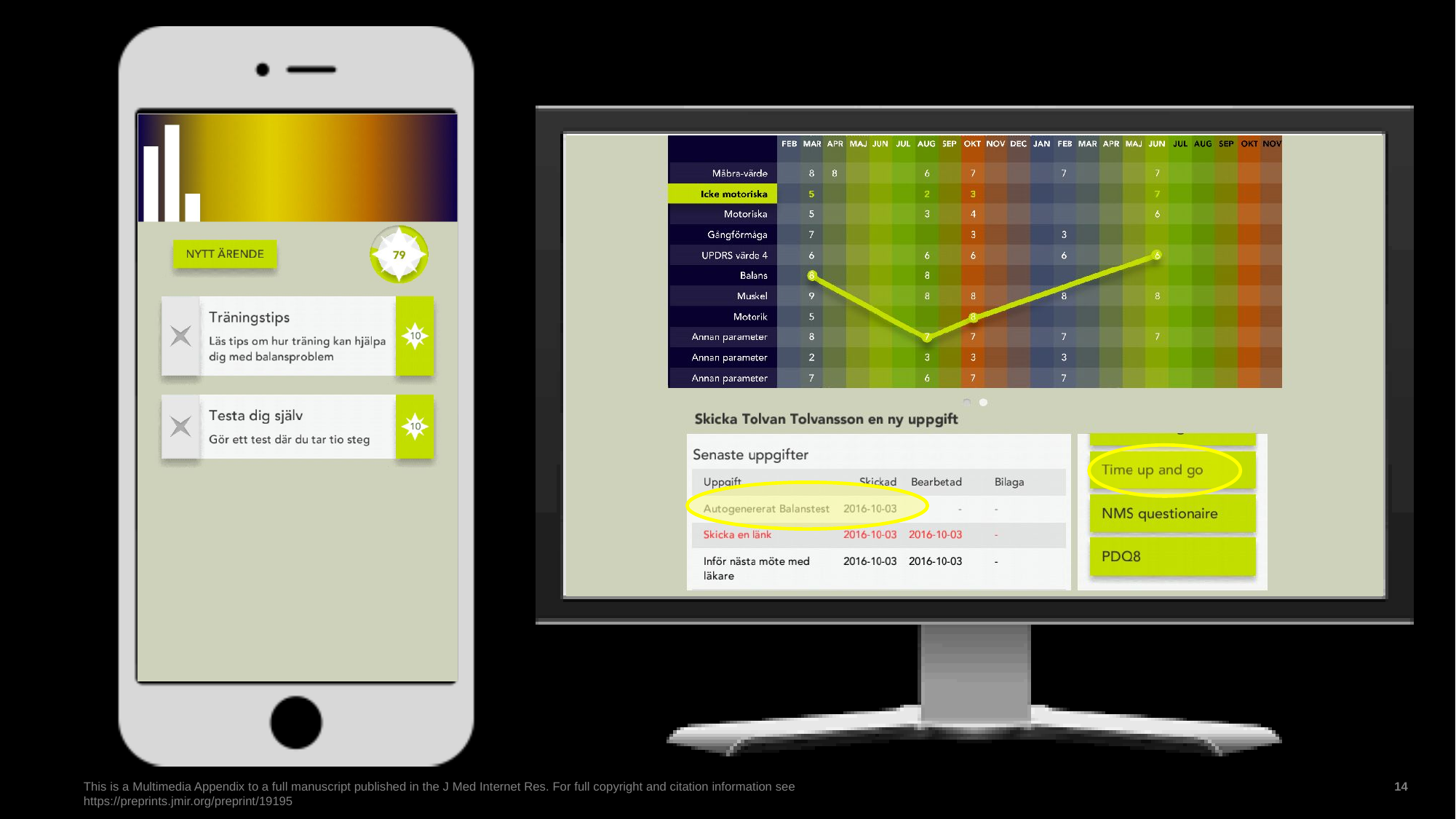

This is a Multimedia Appendix to a full manuscript published in the J Med Internet Res. For full copyright and citation information see https://preprints.jmir.org/preprint/19195
14

## Slide 15
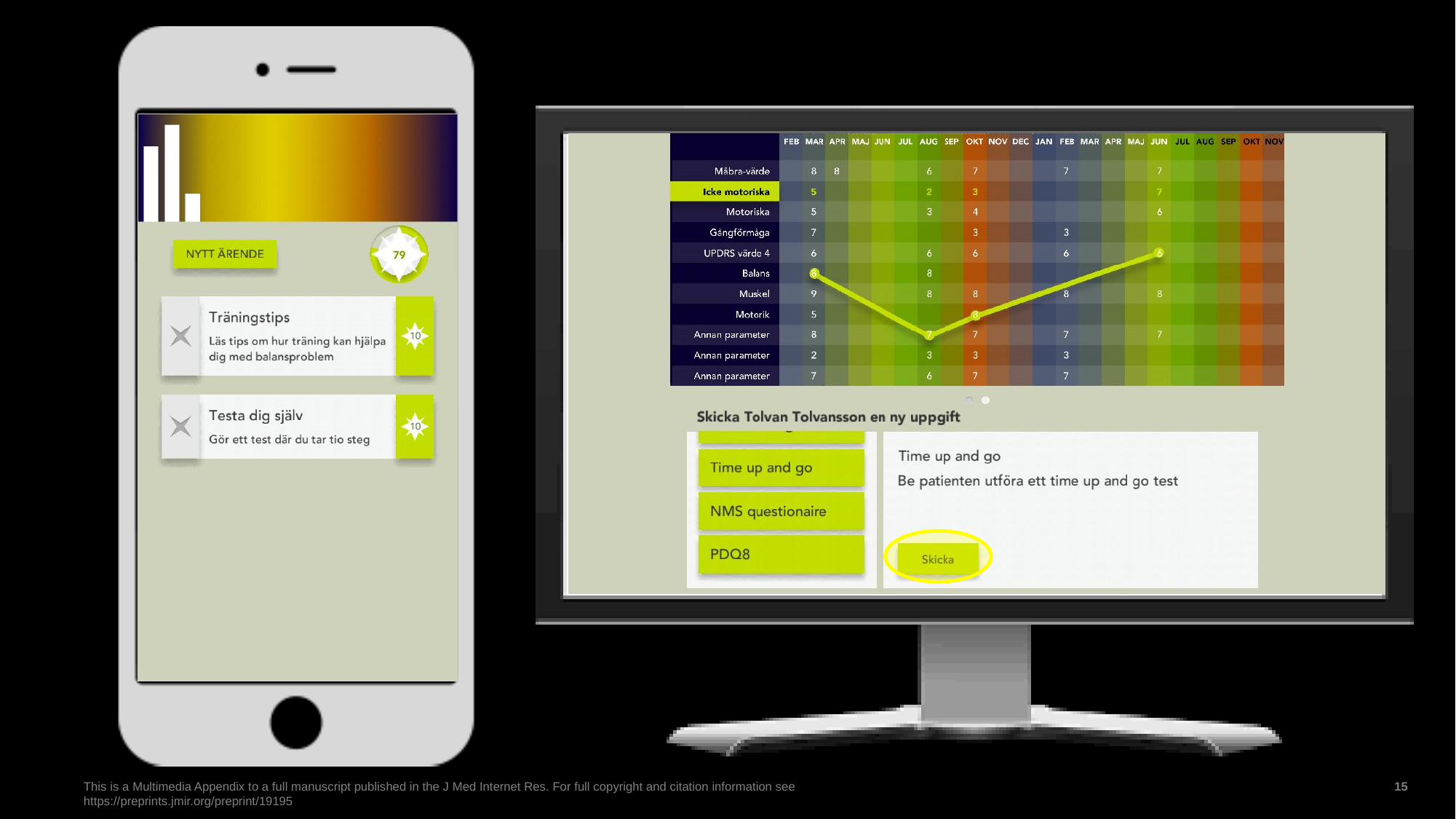

This is a Multimedia Appendix to a full manuscript published in the J Med Internet Res. For full copyright and citation information see https://preprints.jmir.org/preprint/19195
15

## Slide 16
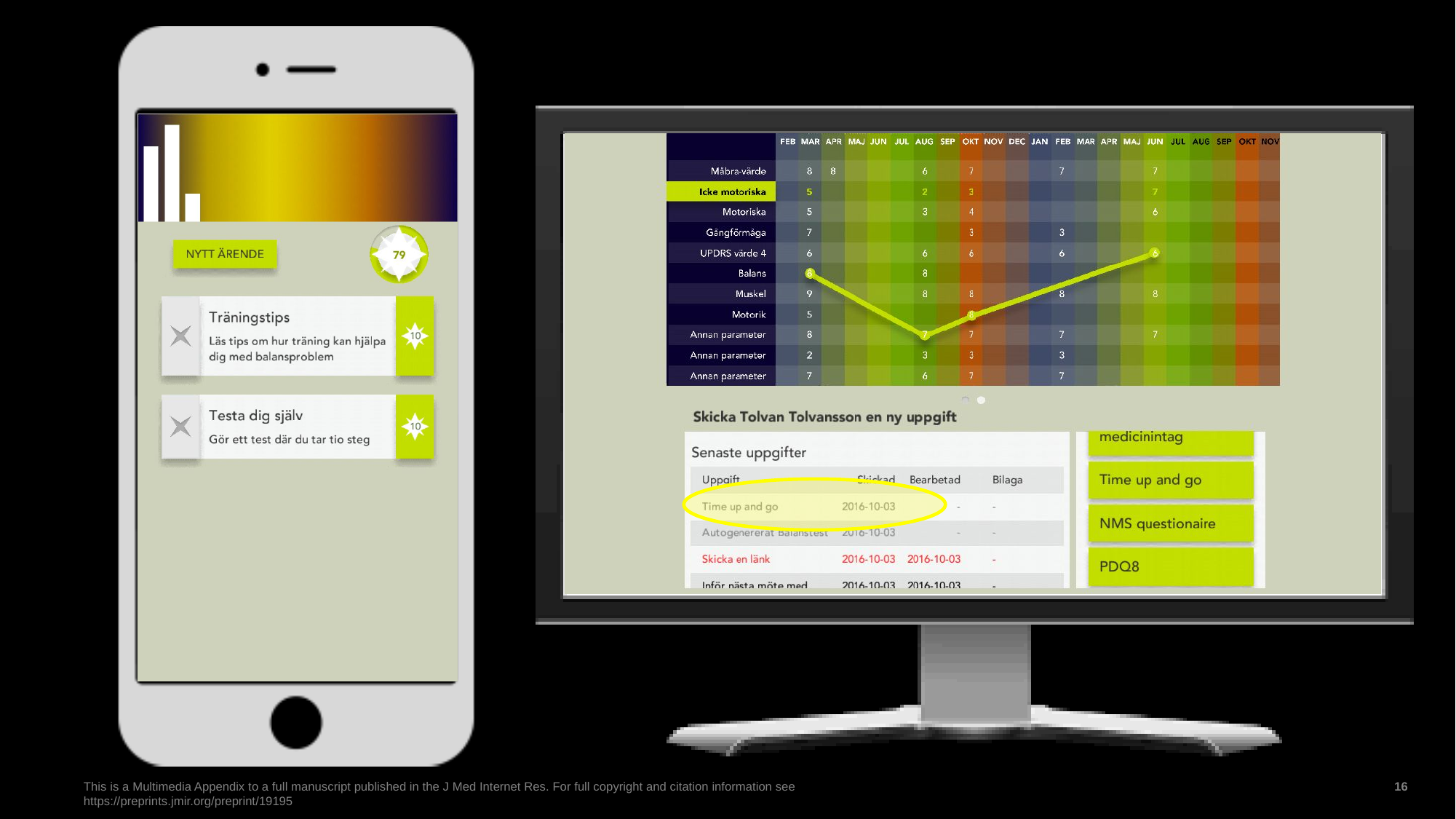

This is a Multimedia Appendix to a full manuscript published in the J Med Internet Res. For full copyright and citation information see https://preprints.jmir.org/preprint/19195
16

## Slide 17
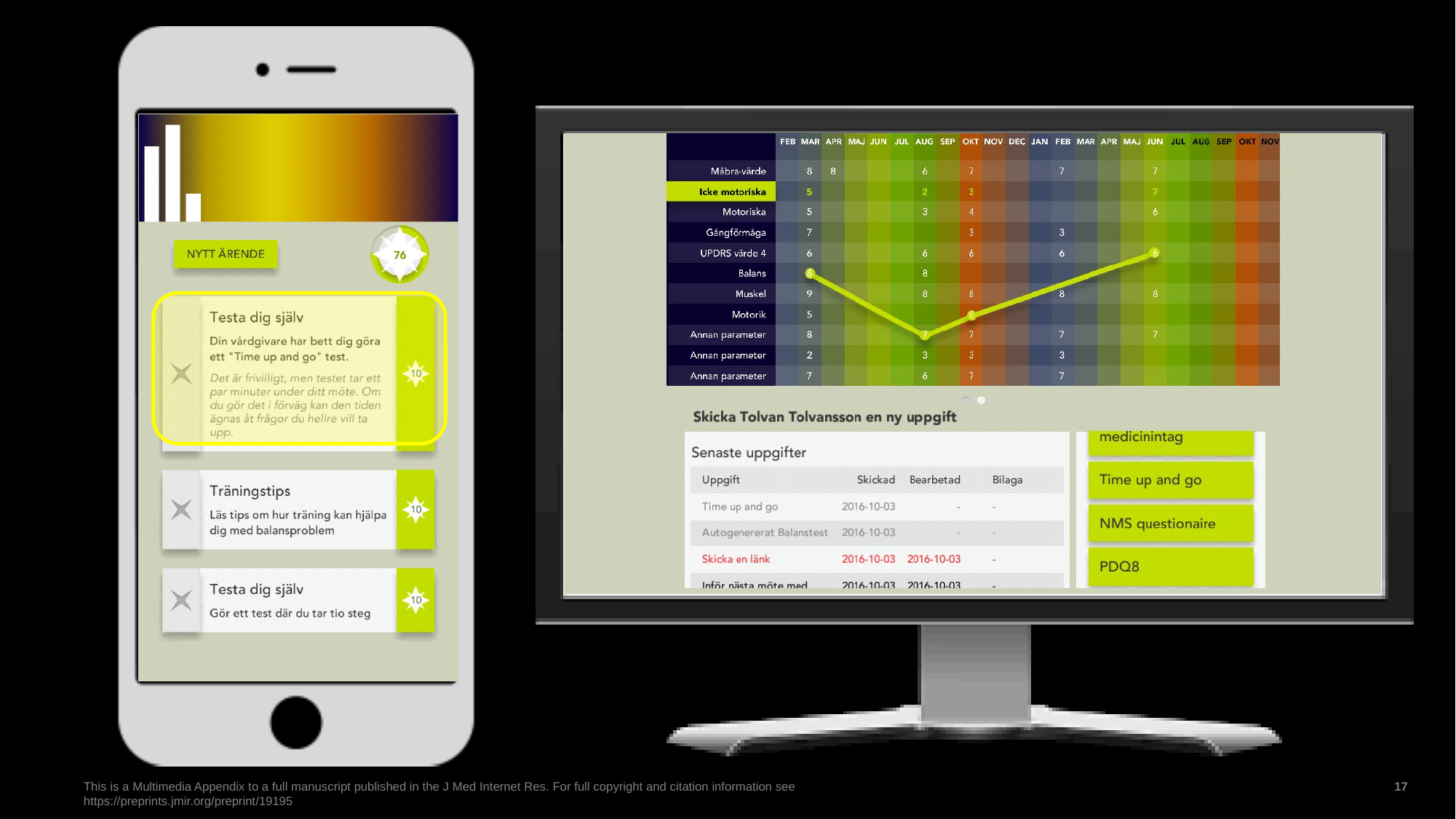

This is a Multimedia Appendix to a full manuscript published in the J Med Internet Res. For full copyright and citation information see https://preprints.jmir.org/preprint/19195
17

## Slide 18
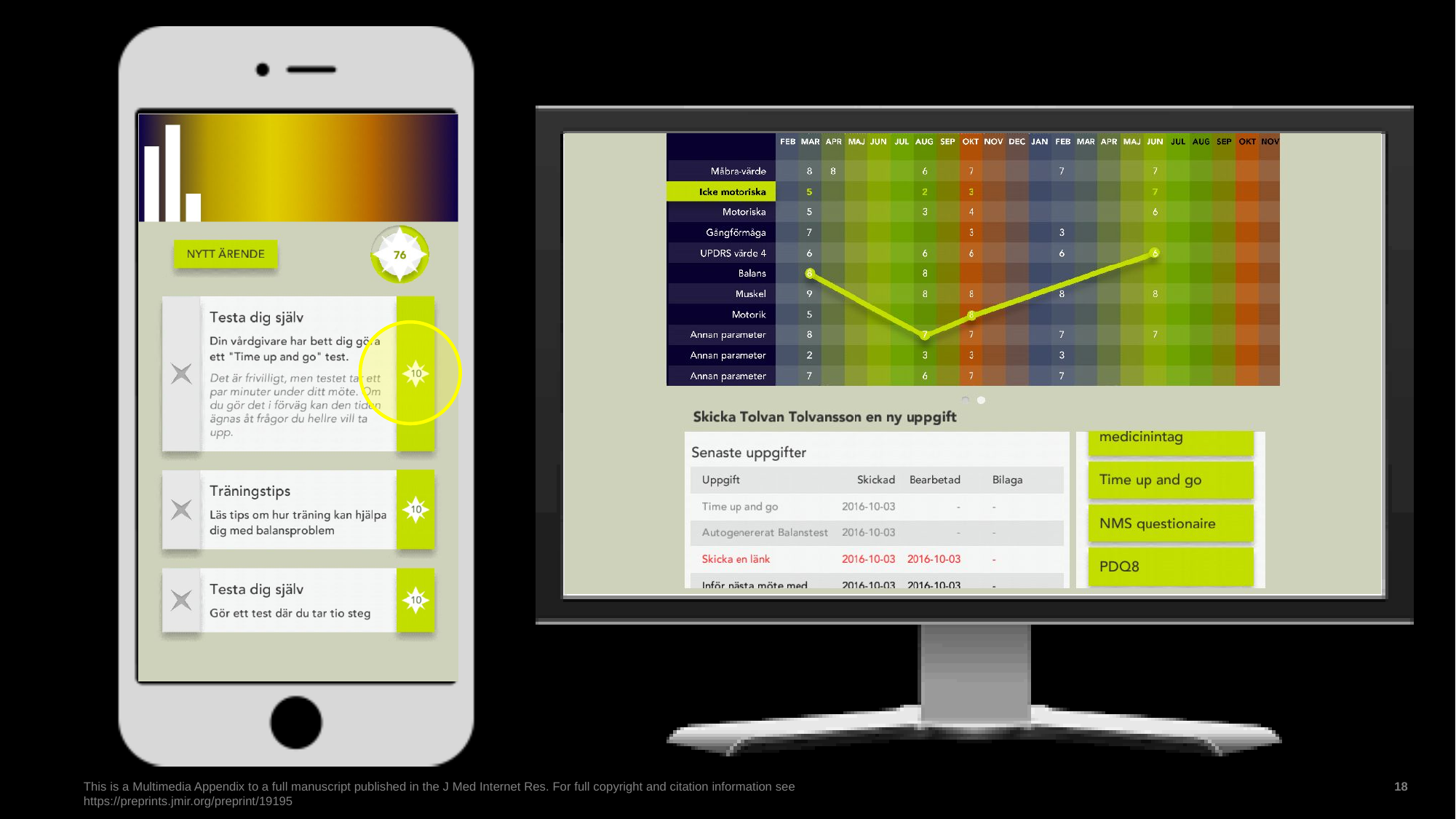

This is a Multimedia Appendix to a full manuscript published in the J Med Internet Res. For full copyright and citation information see https://preprints.jmir.org/preprint/19195
18

## Slide 19
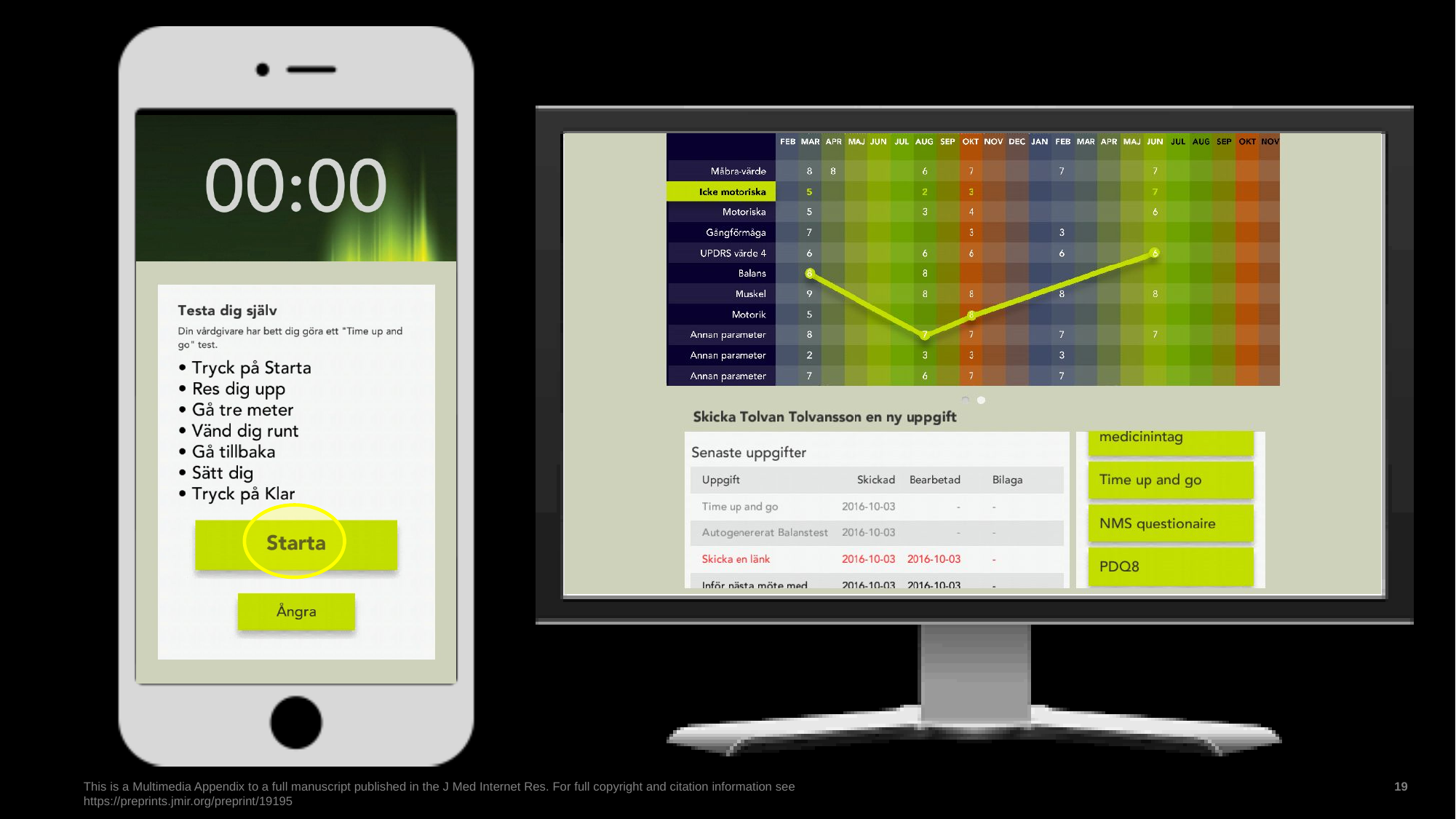

This is a Multimedia Appendix to a full manuscript published in the J Med Internet Res. For full copyright and citation information see https://preprints.jmir.org/preprint/19195
19

## Slide 20
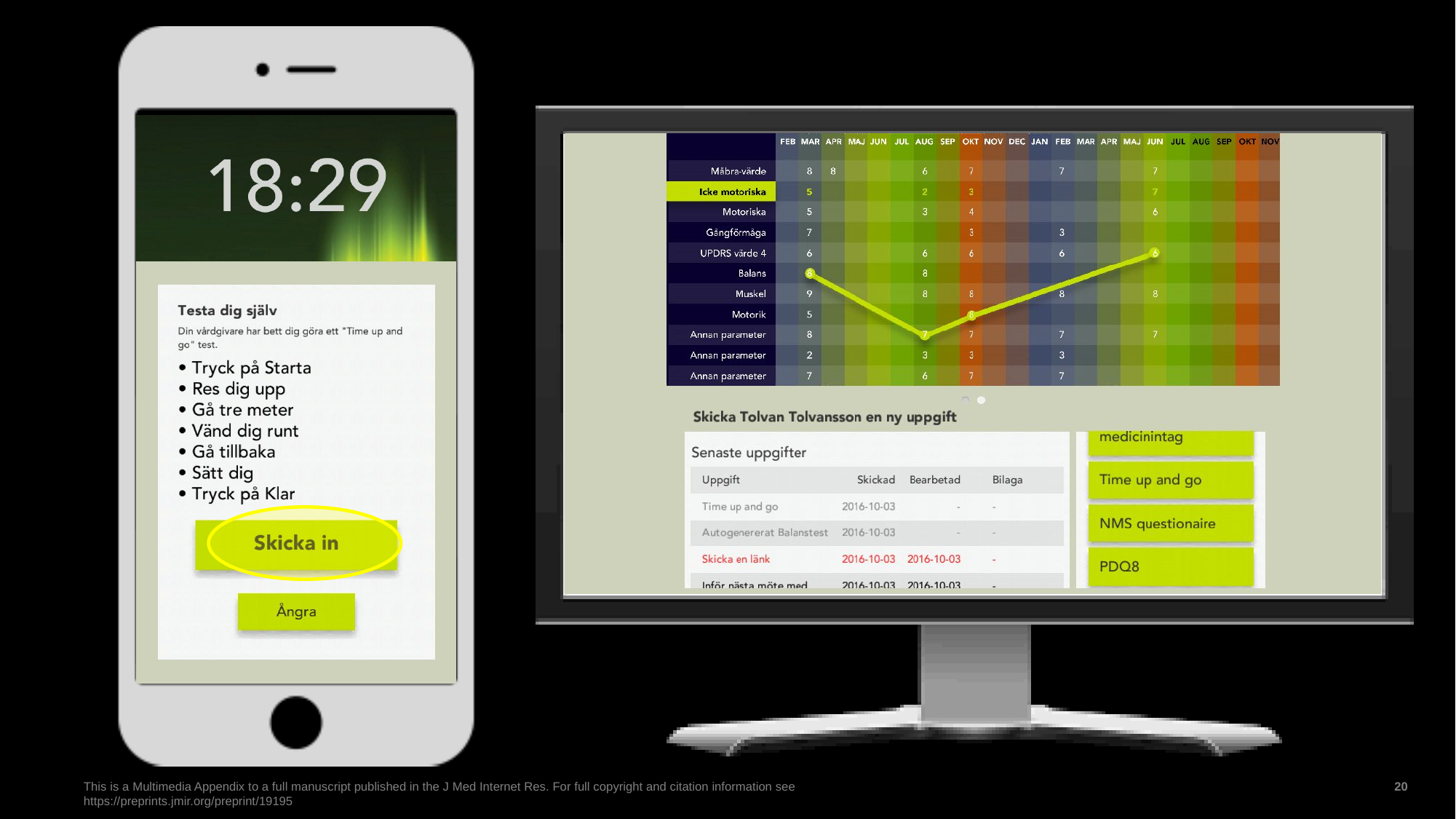

This is a Multimedia Appendix to a full manuscript published in the J Med Internet Res. For full copyright and citation information see https://preprints.jmir.org/preprint/19195
20

## Slide 21
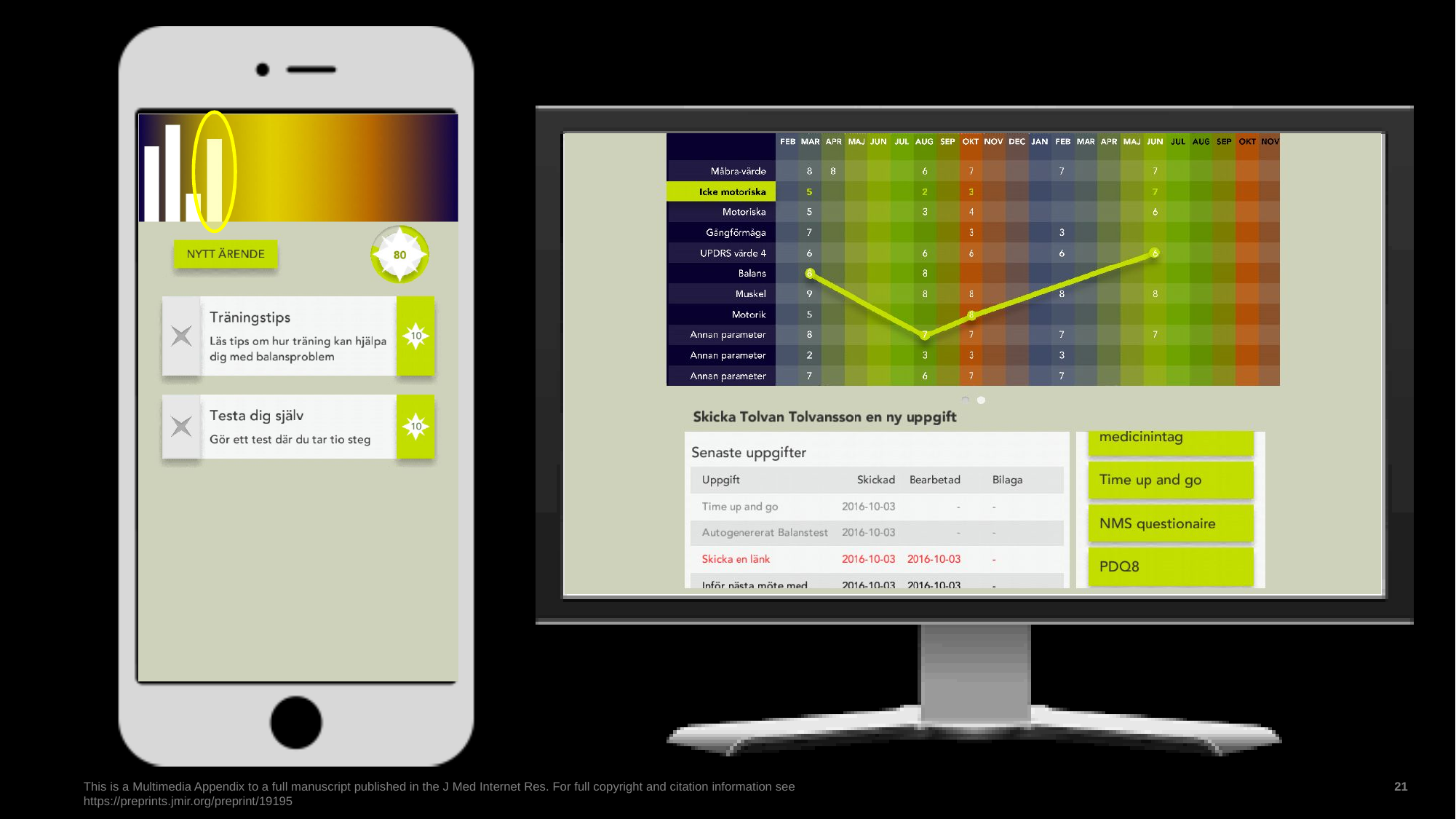

This is a Multimedia Appendix to a full manuscript published in the J Med Internet Res. For full copyright and citation information see https://preprints.jmir.org/preprint/19195
21

## Slide 22
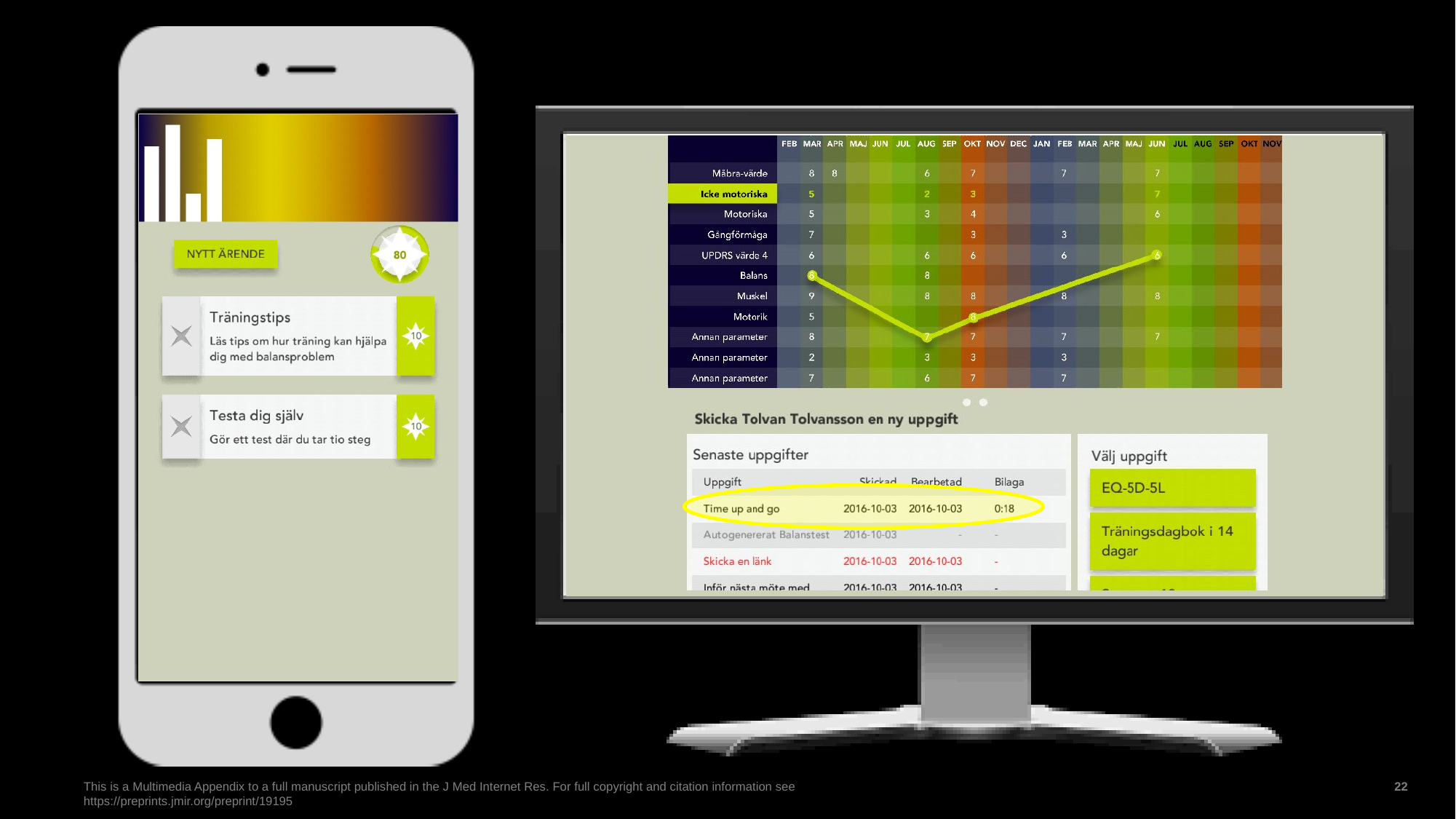

This is a Multimedia Appendix to a full manuscript published in the J Med Internet Res. For full copyright and citation information see https://preprints.jmir.org/preprint/19195
22

## Slide 23
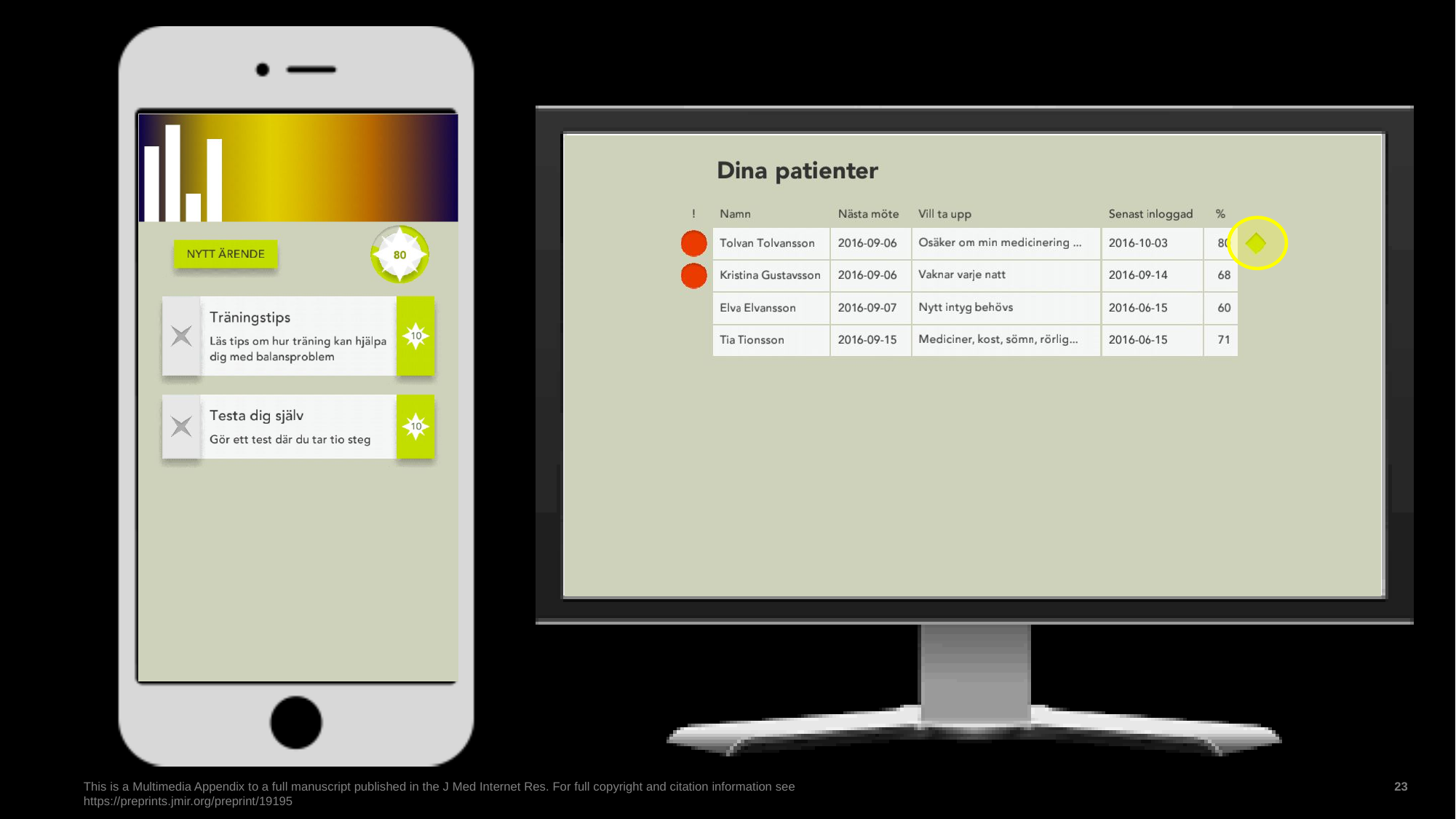

This is a Multimedia Appendix to a full manuscript published in the J Med Internet Res. For full copyright and citation information see https://preprints.jmir.org/preprint/19195
23
