# Supplementary material for: How People with Parkinson's Disease and Health Care Professionals Wish to Partner in Care Using eHealth: Co-Design Study
Source: J Med Internet Res. 2020 Sep 21;22(9):e19195. doi: 10.2196/19195 (PMC7536604; doi:10.2196/19195)
Supplement: Multimedia Appendix 2 [file jmir_v22i9e19195_app2.docx]

# Multimedia Appendix 2 – Descriptive Statistics of the Evaluation Questionnaire

The tables below summarize the results from the evaluation questionnaire.

Table 1. Respondent characteristics.

|  | **PwP** | **Caregivers** | **Total** |
| --- | --- | --- | --- |
| Respondents, n | 31 | 6 | 37 |
| Male, n (column %)  Female, n (column %)  Unknown, n (column %) | 16 (52%)  14 (45%)  1 (3%) | 3 (50%)  3 (50%) | 19 (51%)  17 (46%)  1 (3%) |
| Age in years, median (IQR) | 69 (65-74) | 66 (34-68) | 68 (63-74) |
| Years since diagnosis, median (IQR) | 5 (3-9) | N/A | 5 (3-9) |

Table 2. Rating of the different eHealth functionalities in the co-care prototype.

|  |  | **Descriptive statistics of Likert-type response categories*** | | | | | | | |
| --- | --- | --- | --- | --- | --- | --- | --- | --- | --- |
|  |  | **5** | **4** | **3** | **2** | **1** |  |  |  |
| **Functionality** | **Statement** | **n (%)** | **n (%)** | **n (%)** | **n (%)** | **n (%)** | **Total (N)** | **Mode** |  |
| **Send messages** | 1. I want to be able to receive messages from my healthcare provider, e.g., recommendations or appointment reminders. | 20 (54%) | 14 (38%) | 1 (3%) | 2 (5%) | 0 (0%) | 37 | 5 |  |
| **Send pre-visit message** | 2. I want to be able to send messages to my healthcare provider, e.g., questions about a new health problem. | 29 (78%) | 5 (14%) | 2 (5%) | 1 (3%) | 0 (0%) | 37 | 5 |  |
| **Pre-visit questionnaire** | 3. I want to be able to make notes about questions I want to bring up during the next visit and send these to my healthcare provider in advance. | 26 (72%) | 7 (19%) | 3 (8%) | 0 (0%) | 0 (0%) | 36 | 5 |  |
| **Self-tracking** | 4. I want to be able to fill in forms about my health status, lifestyle etc or make simple self-tests, such as balance tests (and send the results to my healthcare provider using the app). | 16 (43%) | 18 (49%) | 2 (5%) | 0 (0%) | 1 (3%) | 37 | 4 |  |
| **Graphical overview** | 5. I want to be able to keep a diary with medication, physical activity, sleep, etc. (with the option to send information to my healthcare provider using the app). | 20 (54%) | 12 (32%) | 5 (14%) | 0 (0%) | 0 (0%) | 37 | 5 |  |
| **Self-care recommend-ations** | 6. I want to be able to visualize my health status over time to see trends. | 23 (64%) | 12 (33%) | 0 (0%) | 1 (3%) | 0 (0%) | 36 | 5 |  |
| **Receive messages** | 7. I want to be able to receive automatically generated messages (such as links, information about the illness, exercise, sleep) based on questions I send to the healthcare provider or based on my health status. | 16 (44%) | 15 (42%) | 4 (11%) | 1 (3%) | 0 (0%) | 36 | 5 |  |

*: Likert-type response options: 5=Very important, 4, 3=Neutral, 2, 1=Not at all important.

Responses to the questions “New suggestions: ________”

1. *“accumulated statistics”*
2. *“Make sure that [the eHealth service] is synchronized with new models of care”*
3. *“[support for] informal caregivers”*
4. *“I want to be able to fill in all my medications in a form and get alerts/information about expected effects, but the most important is medications that interact negatively”*
5. *“Reminders to take my medication”*

Table 3. Responses to the “Which are the three most important functionalities for you? Fill in the question number (1-7): a. ______ b. ______ c. ______”

|  |  | **Frequencies (n)** | | | | |
| --- | --- | --- | --- | --- | --- | --- |
| **#** | **Functionality** | **a** | **b** | **c** | **Total** |  |
| **1** | Receive messages | 3 | 0 | 2 | 5 |  |
| **2** | Send messages | **10** | 2 | 1 | **13** |  |
| **3** | Send pre-visit message | 2 | **6** | 1 | **9** |  |
| **4** | Pre-visit questionnaire | 3 | 4 | 0 | 7 |  |
| **5** | Self-tracking | 0 | 4 | 5 | **9** |  |
| **6** | Graphical overview | 3 | 5 | **6** | **14** |  |
| **7** | Self-care recommendations | 2 | 1 | 5 | 8 |  |
|  | *Total number of responses* | *23* | *22* | *20* | *65* |  |
